# The mesenteric entry site as a potential weak point in gastrointestinal anastomoses – findings from an *ex-vivo* biomechanical analysis

**DOI:** 10.1007/s00423-024-03318-8

**Published:** 2024-04-13

**Authors:** Kamacay Cira, Saskia Nicole Janett, Carina Micheler, Stephan Heller, Andreas Obermeier, Helmut Friess, Rainer Burgkart, Philipp-Alexander Neumann

**Affiliations:** 1 Department of Surgery, Klinikum Rechts Der Isar, TUM School of Medicine and Health, Technical University of Munich, 81675 Munich, Bavaria Germany; 2 Department of Orthopaedics and Sports Orthopaedics, Klinikum Rechts Der Isar, TUM School of Medicine and Health, Technical University of Munich, Munich, Germany; 3 Institute for Machine Tools and Industrial Management, TUM School of Engineering and Design, Technical University of Munich, Munich, Germany

**Keywords:** Anastomotic leakage, Biomechanics, Anastomosis, Mesentery, Bursting pressure

## Abstract

**Purpose:**

Gastrointestinal disorders frequently necessitate surgery involving intestinal resection and anastomosis formation, potentially leading to severe complications like anastomotic leakage (AL) which is associated with increased morbidity, mortality, and adverse oncologic outcomes. While extensive research has explored the biology of anastomotic healing, there is limited understanding of the biomechanical properties of gastrointestinal anastomoses, which was aimed to be unraveled in this study.

**Methods:**

An *ex-vivo* model was developed for the biomechanical analysis of 32 handsewn porcine end-to-end anastomoses, using interrupted and continuous suture techniques subjected to different flow models. While multiple cameras captured different angles of the anastomosis, comprehensive data recording of pressure, time, and temperature was performed simultaneously. Special focus was laid on monitoring time, location and pressure of anastomotic leakage (LP) and bursting pressures (BP) depending on suture techniques and flow models.

**Results:**

Significant differences in LP, BP, and time intervals were observed based on the flow model but not on the suture techniques applied. Interestingly, anastomoses at the insertion site of the mesentery exhibited significantly higher rates of leakage and bursting compared to other sections of the anastomosis.

**Conclusion:**

The developed *ex-vivo* model facilitated comparable, reproducible, and user-independent biomechanical analyses. Assessing biomechanical properties of anastomoses offers an advantage in identifying technical weak points to refine surgical techniques, potentially reducing complications like AL. The results indicate that mesenteric insertion serves as a potential weak spot for AL, warranting further investigations and refinements in surgical techniques to optimize outcomes in this critical area of anastomotic procedures.

**Supplementary Information:**

The online version contains supplementary material available at 10.1007/s00423-024-03318-8.

## Introduction

Anastomotic leakage (AL) is still a significant complication [1–13] that can lead to severe infections and even life-threatening sepsis [14, 15]. Despite ongoing advancements in surgical techniques and perioperative treatments, AL presents with varying rates depending on the location of the intestinal anastomosis with up to 19.5% [6, 12] in the upper gastrointestinal tract and up to 25.6% in the lower gastrointestinal tract [3, 5, 7]. Resulting mortality rates range from 4.3% to 43.8% [6, 16–18]. Furthermore AL has been linked to local [19] and distant tumor recurrence [20] in patients with gastrointestinal malignancies. AL causes not only significant personal suffering for patients but also substantial economic challenges for healthcare systems [21].

Therefore, it is not surprising that investigation of anastomotic healing and prevention of AL have been of central importance in surgical research to date. Risk factors for AL can be broadly classified into surgical-related and patient-related factors. The former encompasses errors in surgical techniques, such as anastomosis under tension, inadequate anastomotic perfusion, and suboptimal technique, both in the context of laparoscopic and open surgery. Additional considerations involve the timing of surgery, surgeon experience, and the choice between handsewn or stapled procedures, as well as the anastomotic level or the use of a protective stoma. On the other hand, patient-related factors present a distinct set of considerations, including obesity, malignancy, poor nutritional status, smoking, blood loss, chemoradiotherapy, and the composition of the gut microbiome [22, 23]. Understanding the interplay between these factors is crucial for comprehensive risk assessment and effective prevention strategies in the context of anastomotic healing. While these categorizations offer a comprehensive overview of various risk factors associated with AL, it is crucial to underscore the significance of biomechanical studies.

The biomechanical intricacies of anastomoses play a pivotal role in the healing process. A profound comprehension of how mechanical forces, suture techniques, and anatomical considerations interact is imperative for successful outcomes. Without meticulous attention to these biomechanical aspects, an environment conducive to adequate wound healing during and after surgery cannot be established. Indeed, the effectiveness of prevention strategies and the overall success of anastomotic procedures hinge upon the surgeon's ability to apply sound biomechanical principles during and after surgery. The selection of appropriate suture techniques, consideration of mechanical stress, and the careful evaluation of anatomical vulnerabilities are integral components of this biomechanical understanding. Biomechanical studies involve quantitative measurements of factors such as bursting pressure (BP), tensile strength, suture holding capacity, and other mechanical properties and provide valuable insights into identifying potential risk factors, developing preventive strategies, and aiding surgeons in making informed decisions on anastomotic technique [24–27].

The objective of this study was to investigate the biomechanical properties of gastrointestinal anastomoses, with a specific emphasis on analyzing the time, location, and pressure of leakage and bursting. The aim was twofold: firstly, to identify technical weak points within the anastomosis, and secondly, to assess whether variations in suture techniques and flow models would influence these weak points. This was achieved through a comprehensive *ex-vivo* test setup, specifically developed for this study.

## Materials and methods

### Chemicals and consumable materials

A detailed list of used chemicals, reagents, parent solutions, surgical and consumable material can be found in Supplementary Table 1.

### Biologic material – porcine small intestine

The resemblance between porcine small intestine and its human counterpart, both at microscopic and macroscopic levels, has been well-documented in prior research [28, 29]. Thus, for all experiments conducted in this study, porcine small intestine was selected for anastomosis formation. The choice of the origin of the tissue utilized in the experiments was guided by ethical considerations, including 3 R principles for reduction, replacement and refinement of animal experiments [30]. Thus the porcine small intestine was obtained from the Center of Preclinical Research affiliated with the Klinikum rechts der Isar of the Technical University of Munich. Therefore, the utilization of animal products in this study solely relied on byproducts from animals already sacrificed for other experimental purposes, eliminating the need to sacrifice additional animals specifically for the experimental purpose in this study.

The porcine small intestine was obtained promptly after the scarification of the animals and underwent a thorough cleaning process using water before being utilized. To counteract the potential adverse effects of tissue degeneration on experimental outcomes, the harvested tissue was stored in a refrigerator, maintained at a temperature of 4 degrees Celsius (°C), and kept moisturized for a maximum of 12 h.

### Ex-vivo model for evaluation of stability and pressure resistance of gastrointestinal anastomoses

**Fig. 1:**
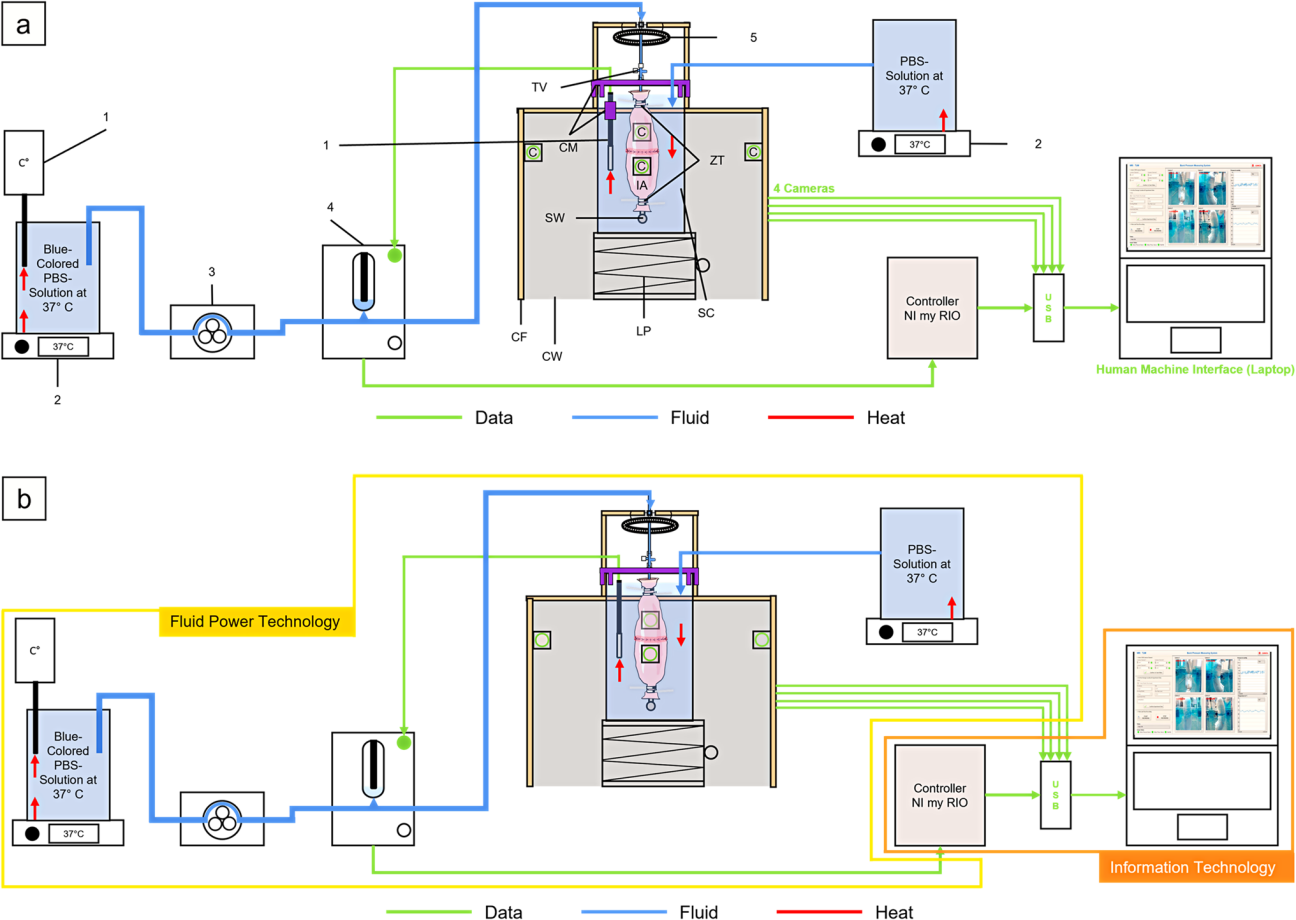

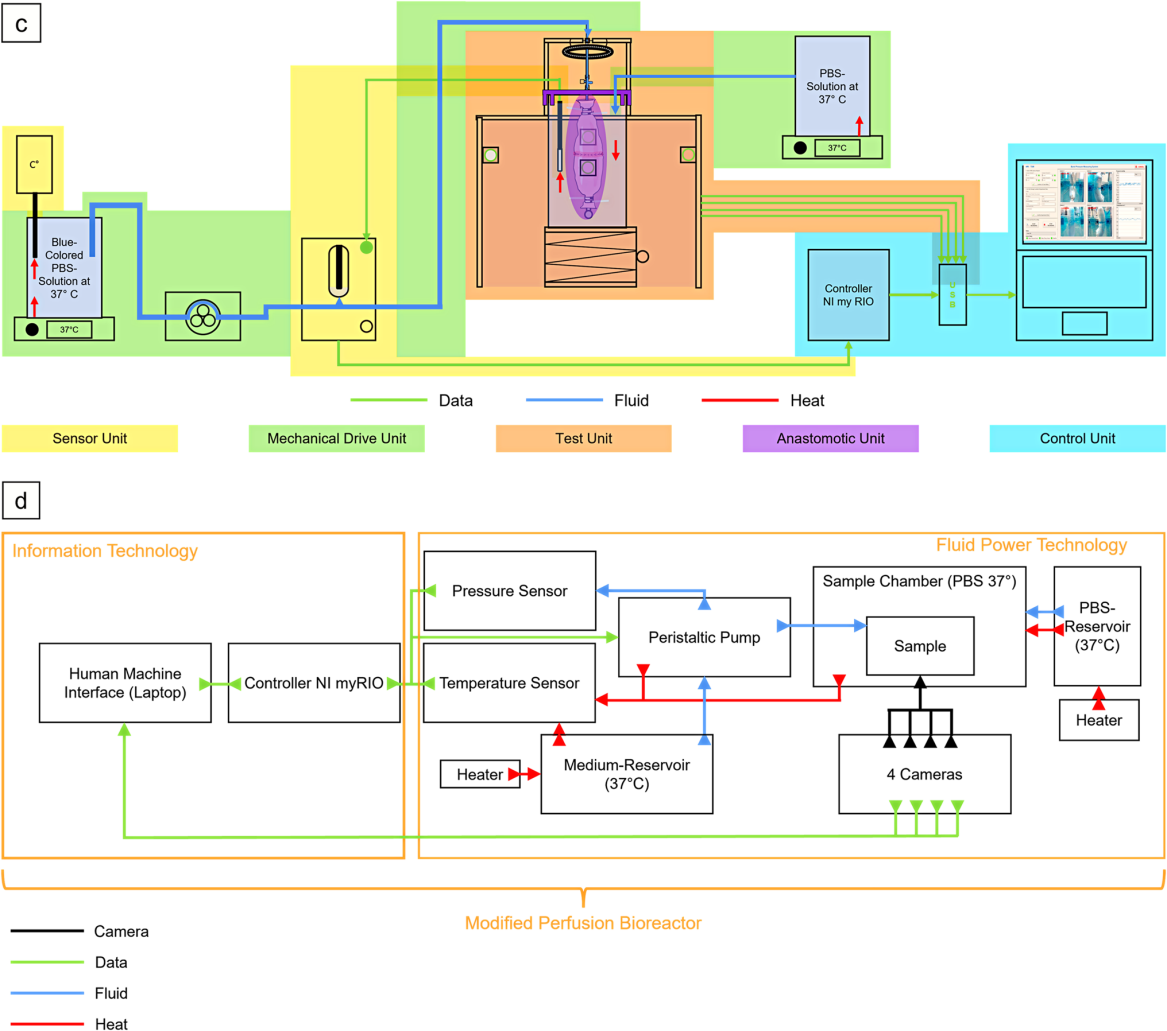
Schematic representation of innovative *ex-vivo* model for evaluation of stability and pressure resistance of gastrointestinal anastomoses. (**a**) Schematic representation of the experimental setup illustrating the key components in the test configuration. (**b**) Schematic representation of the experimental setup categorized into two technologies: information technology and fluid power technology. (**c**) Schematic representation of the main units of the experimental setup: sensor unit; mechanical drive unit; test unit; anastomotic unit; control unit. (**d**) Modified perfusion bioreactor. Process description of the modified perfusion bioreactor, encompassing both information technology and fluid power technology. C = Camera; CF = Custom-made aluminum square shaped frame; CM = Custom-made 3D-printed stabilization brackets; CW = Custom-made plastic walls with cutouts; IA = Intestinal anastomosis; LP = Laboratory lifting platform; SC = Sample chamber; SW = Stainless steel screw; TV = Three-way-valve; ZT = Zip ties; 1 = Temperature sensor; 2 = Heater; 3 = Peristaltic pump; 4 = Pressure sensor; 5 = LED Ring light. [31–33] (Modified from Micheler 2018a and Micheler, Geck, Charitou et al., Curr. Dir. Biomed. Eng 2021)

**Fig. 2:**
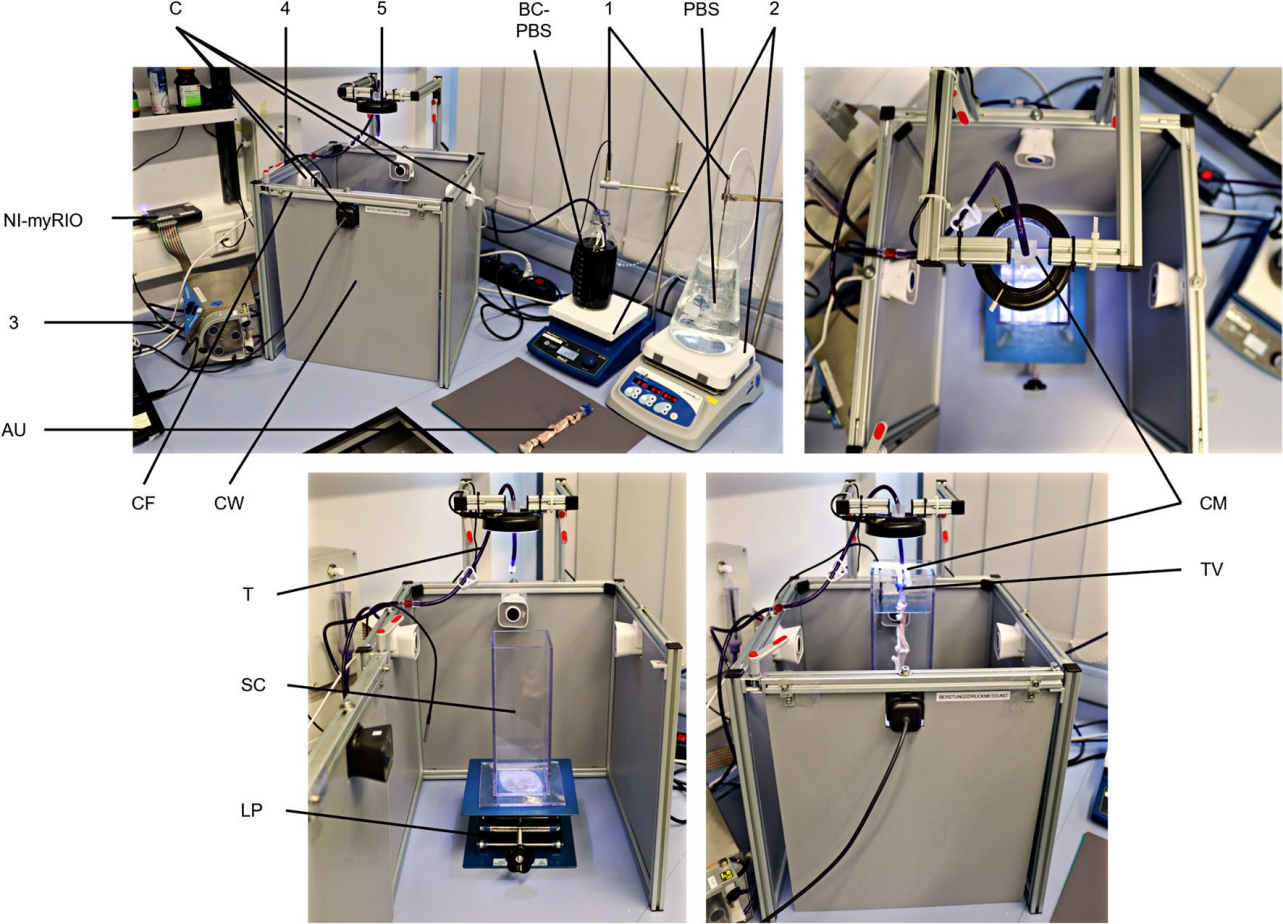
Innovative *ex-vivo* model for evaluation of stability and pressure resistance of gastrointestinal anastomoses. BC-PBS = Blue-colored PBS-solution at 37 °C; C = Camera; CF = Custom-made aluminum square shaped frame; CM = Custom made 3D-printed stabilization brackets; CW = Custom-made plastic walls with cutouts; LP = Laboratory lifting platform; NI-myRIO = NI-myRIO controller; PBS = PBS-solution at 37 °C; SC = Sample chamber; TV = Three-way-valve; T = Tube; ZT = Zip ties; 1 = Temperature sensor; 2 = Heater; 3 = Peristaltic pump; 4 = Pressure sensor; 5 = LED Ring light

**Fig. 3:**
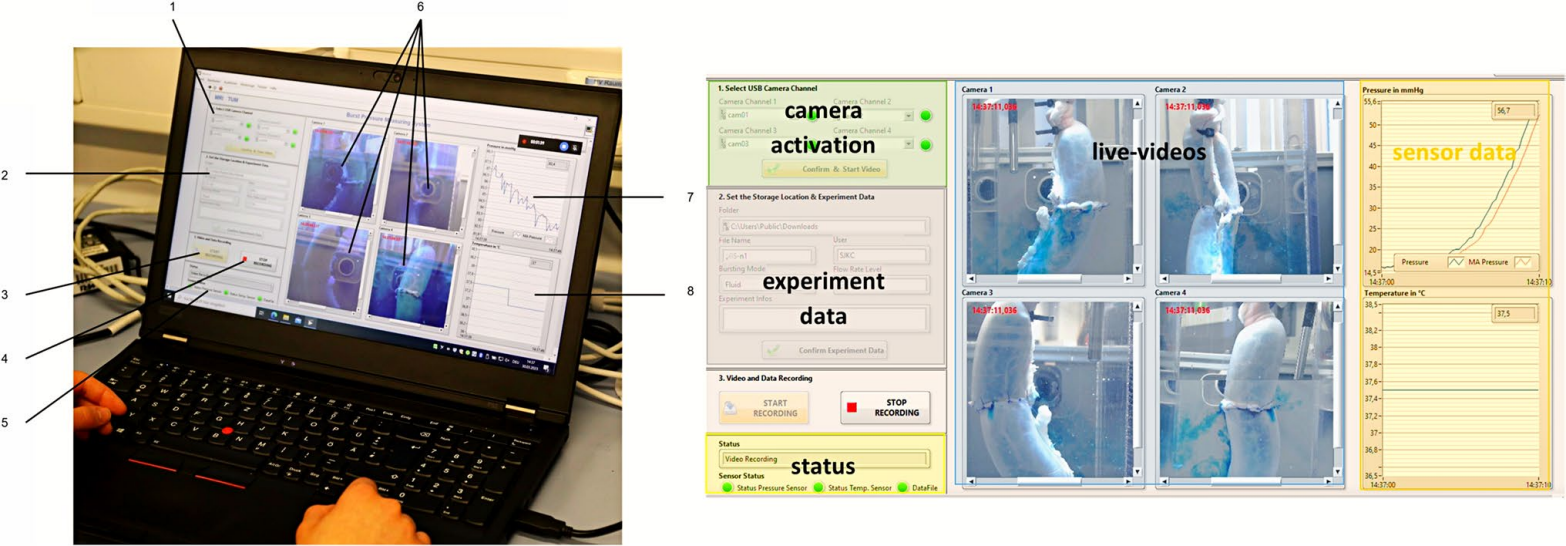
Human machine interface (computer) during an experiment. The figure displays the HMI on a computer used during the experiment. 1 = Camera activation: allows the user to activate the cameras for recording; 2 = Enter experimental data: enables inputting relevant experimental data; 3 = Start recording of the experiment: initiates the recording of the experiment; 4 = Stop recording of the experiment: halts the recording process; 5 = Video and sensor status: provides information on the status of videos and sensors; 6 = Four cameras capturing the anastomosis: shows the real-time footage from the four cameras capturing the anastomosis during the experiment; 7 = Pressure measurement: displays the intraluminal pressure

The *ex-vivo* test setup consists of five main components: the anastomotic unit, the test unit, the sensor unit, the mechanical drive unit, and the control unit (Fig. 1a and c; Fig. 2). Based on the perfusion bioreactor developed by Micheler et al. [31–33] the integrated system can be further categorized into two technologies: information technology and fluid power technology (Fig. 1a, b and d). The key components of the integrated system in this model include a human machine interface (HMI) (Fig. 3), a controller, actuators, sensors, a fluid system, and a sample chamber. (Fig. 1a, b and d; Fig. 2; Supplementary Fig. 1; Supplementary Fig. 2) The two technologies and main components of the *ex-vivo* system are detailed in Supplementary Table 2.

#### Flow rate models

Two flow rate models were employed to simulate variations in fluid flow rates in the small intestine under different physiological conditions. Studies have shown that the fluid flow rate in the proximal small intestine is approximately 2.5 mL/minute (min) in fasting conditions [34, 35] and 20 mL/min after meals [36–40]. While the resting intraluminal pressure in the small intestine varies among the literature (6–13 mm of mercury (mmHg) [41–43] up to 20–30 mmHg during various peristaltic activities [44]), a sudden increase in intraabdominal and therefore intraluminal pressure can occur with activities, such as during Valsalva maneuvers (up to 40 mmHg), coughing (up to 100 mmHg), or jumping (up to 170 mmHg) [43, 45].

The low flow (LF) model with a fluid flow rate of 20 mL/min was utilized to simulate a physiological increase in intraluminal intestinal pressure under normal conditions (moderate increase in fluid influx, simulating the rise of intraabdominal pressure during regular activities).

The high flow (HF) model with a fluid flow rate of 200 mL/min was used to simulate a sudden increase in intraluminal intestinal pressure such as with coughing or jumping (substantial surge in fluid influx, simulating the abrupt elevation in intraabdominal pressure).

These two models aimed to replicate changes in the intraabdominal and therefore intraluminal pressure observed during different physiological states, providing a basis for studying the effects of pressure on anastomotic stability and pressure resistance.

#### Sample preparation and anastomotic techniques

Supplementary Fig. 3 illustrates the instruments and materials required to create the anastomotic unit.

The porcine small intestines were dissected into 20 cm (cm) long segments (Supplementary Fig. 4.a) and rehydrated at 37 °C before performing the intestinal anastomosis. Rehydration was performed with modification to the method described by Daristotle et al. [46] and involved submerging the segments in phosphate buffered saline solution preheated to 37 °C for five min. (Supplementary Fig. 4.a and 4.b).

For the feasibility trial, 32 handsewn sufficient end-to-end anastomoses were performed by one investigator (Kamacay Cira (K.C.)) on the porcine small intestine (cadaver of two pigs) in a random order, using either an interrupted single button suture (SBS) technique (n = 16) (Supplementary Fig. 5) or continuous suture (CS) technique (n = 16) (Supplementary Fig. 6). The suture material utilized for all anastomoses was 4–0 Polydioxanone. While only one surgeon conducted the anastomoses to minimize variability, another surgeon (Philipp-Alexander Neumann (P-A.N.), a senior surgeon) supervised the performance of the anastomoses to eliminate systematic technical errors. The anastomoses were further divided randomly by another investigator (Saskia Nicole Janett (S.N.J.)) into the following four experimental series:

- a) *Experimental series 1 (SBS-LF)*: eight handsewn sufficient small intestinal SBS end-to-end anastomoses were created. (Supplementary Figure 5) These anastomoses were subsequently tested in the LF model;
- b) *Experimental series 2 (SBS-HF)*: eight handsewn sufficient small intestinal SBS end-to-end anastomoses were created. (Supplementary Figure 5) These anastomoses were subsequently tested in the HF model;
- c) *Experimental series 3 (CS-LF)*: eight handsewn sufficient small intestinal CS end-to-end anastomoses were created. (Supplementary Figure 6) These anastomoses were subsequently tested in the LF model;
- d) *Experimental series 4 (CS-HF)*: eight handsewn sufficient small intestinal CS end-to-end anastomoses were created. (Supplementary Figure 6) These anastomoses were subsequently tested in the HF model.

### Data analysis

**Fig. 4:**
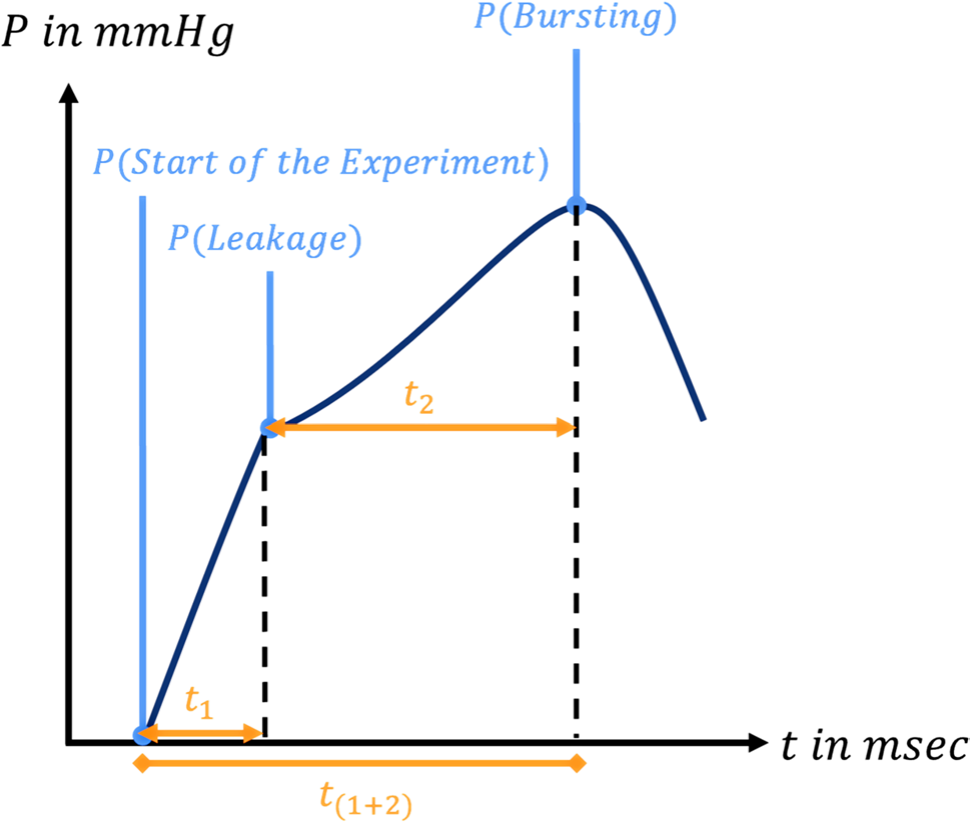
Analysis of key parameters during the experimental process. This figure presents key parameters of the experimental process, including start pressure, LP, BP, and time intervals. mmHg = millimeters of mercury; msec = millisecond; P = Pressure; t = time; t_1_ = Start to LP Time; t_2_ = LP to BP; Time; t _(1+2)_ = Start to BP Time

Detailed data analysis is presented in Supplementary Table 3. Key parameters, including start pressure, LP, BP and various time intervals, were quantitatively studied to assess anastomotic performance (Fig. 4). These measurements prove insights into anastomotic integrity and endurance under different intraluminal pressures, simulating e.g. physiological and forceful expiratory activities. For more detailed information on the definition of different key parameters and a description of data acquisition procedures, please refer to Supplementary Table 4. Interrelated analyses were conducted on the outcomes of different experimental series to understand the temporal relationship between LP and BP, as well as the pressure and time differences associated with these events. More information about parameter definitions, data acquisition procedures, and their significance can be found in Supplementary Table 5.

#### Leakage and bursting location analysis

**Fig. 5:**
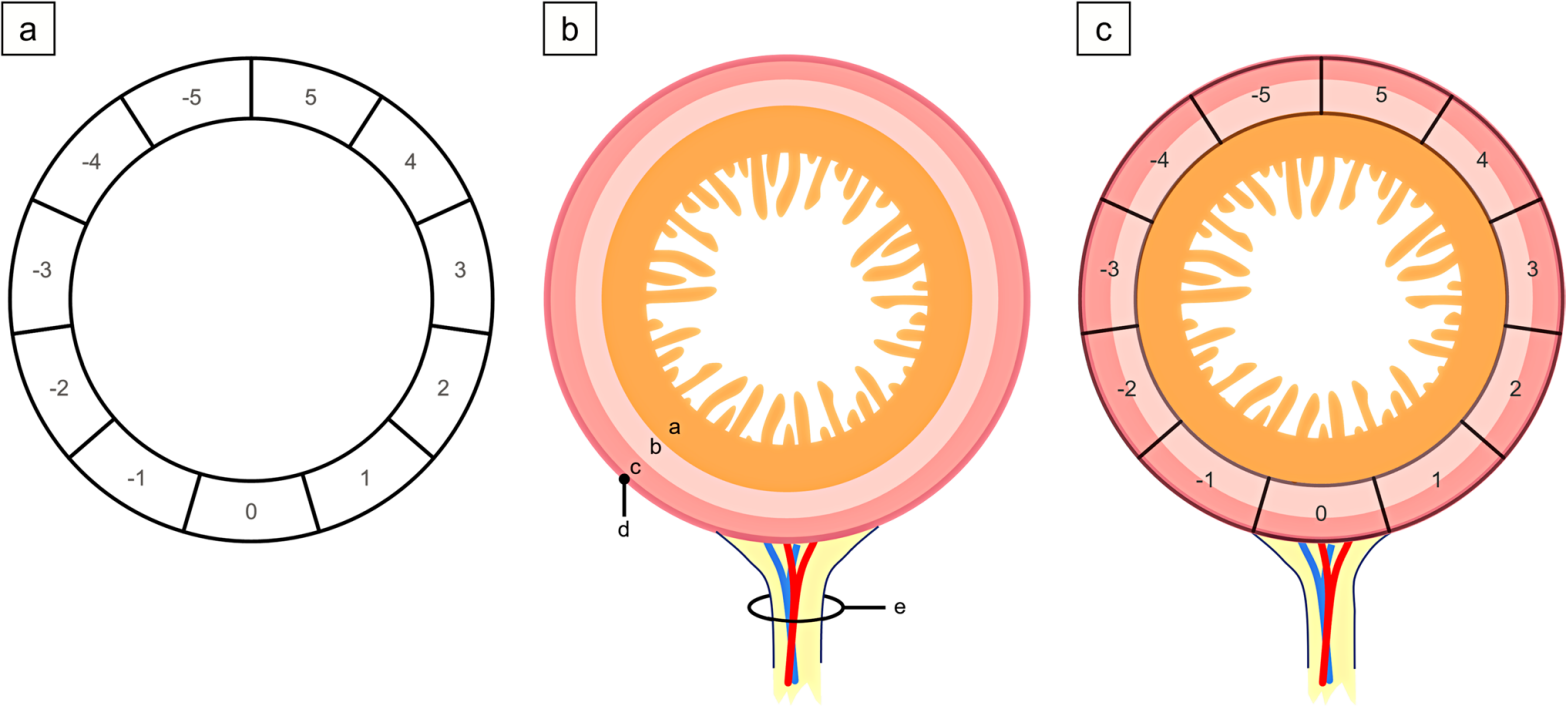
Location analysis of anastomotic leakage and bursting in small intestinal anastomoses. (a) The circular section model depicts the anastomoses partitioned into eleven equidistant sections, designated by numbers ranging from -5 to 5. The reference point, 0 (or M0), precisely indicates the location of the mesenteric attachment to the intestine. (b) Schematic cross-section of the small intestine. (c) The composite presentation of (a) and (b) provides insights into the spatial distribution of the eleven equidistant sections. a = Tunica mucosa; b = Tunica submucosa; c = Tunica muscularis; d = Tunica serosa; e = Mesentery

**Fig. 6:**
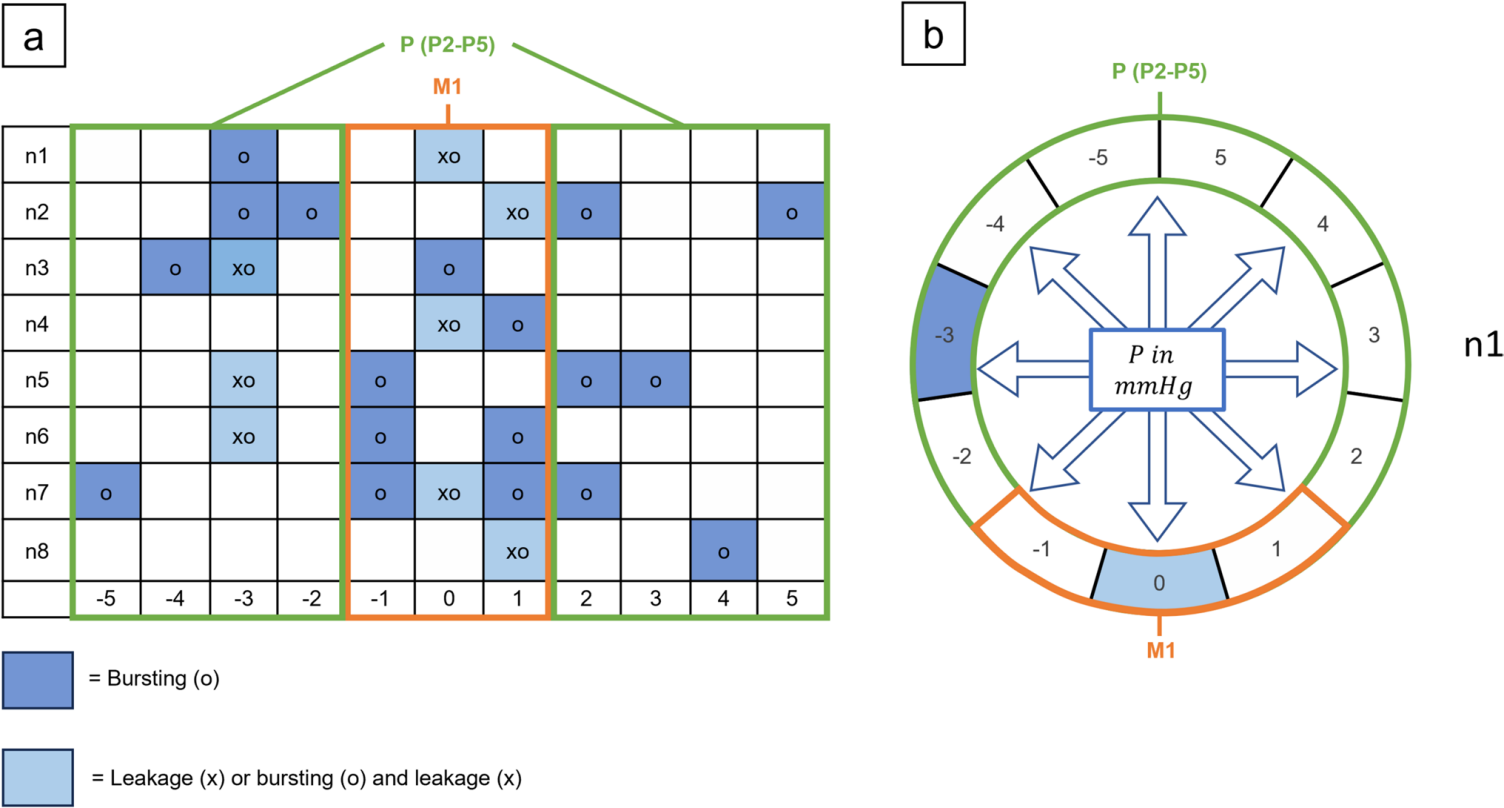
Location analysis: Spatial distribution of anastomotic leakage and bursting in small intestinal anastomoses. (a) To simplify data evaluation and presentation, the circular sections were transformed into a linear grid, with 0 at the center, -5 to the far left, and 5 to the far right. The grid was further divided into two zones: the mesenteric zone (between -1 and + 1, labeled as zone M1) and the peripheral zone (ranging from -2 to -5 and + 2 to + 5, labeled as zone P (P2-P5)). Leakage points are marked with "x" and bursting points with "o". (b) The circular section model represents n1 on the grid

In order to investigate the location of leakage and bursting of the porcine small intestinal anastomoses, images from all four cameras capturing different angles of the anastomosis were correlated with the corresponding measured pressures for each experimental trial. The anastomosis was divided into eleven equal sections, numbered from -5 to 5, with 0 (or M0) representing the precise location of mesenteric attachment to the intestine (Figs. 5 and 6).

To simplify data evaluation and presentation, these circular sections were transformed into a linear grid, with 0 positioned at the center, -5 to the far left, and 5 to the far right. The grid was further divided into two zones: the mesenteric zone (between -1 and + 1) and the peripheral zone (ranging from -2 to -5 and + 2 to + 5), labeled as zones M1 and P (P2 – P5) respectively (Fig. 6).

After marking the locations of leakage and bursting based on the corresponding images of each anastomosis, the number of occurrences per zone was assessed for each experimental trial. Comparisons were made to explore the occurrence of leakage and bursting between the mesenteric and peripheral zones within each experimental series and across different experimental groups. Additionally, a general comparison was performed to determine if leakage and bursting tended to manifest more frequently in either the mesenteric or peripheral zone across all experimental groups (Fig. 6).

### Statistical analysis

Outcomes with a *p*-value of ≤ 0.05 in this study are considered as significant. Microsoft Excel and GraphPad Prism were used for statistical analyses. Descriptive statistics were computed, providing various parameters like mean, standard error (*SEM*), median, standard deviation (*SD*), sample variance, kurtosis, skewness, range (minimum, maximum), sum, count, and a 95% confidence level with upper and lower limits. The upper and lower confidence intervals (*CI*) were calculated for comparisons within experimental series. To compare the results of the descriptive statistical analysis among the experimental series, the Mann–Whitney U test [47, 48], a non-parametric test suitable for comparing two independent groups, especially when data isn't normally distributed or sample sizes are small, was employed. The Fisher's exact test [49], a statistical tool suitable for analyzing categorical data with small sample sizes, was employed to analyze and compare the incidence of leakage and bursting within and between areas of the anastomosis – specifically, the mesenteric zone and peripheral zone – across each of the four experimental groups.

The Odds ratio (*OR)* was used in this study to assess the association and quantify the strength of relationships between different aspects of anastomotic performance, with a particular focus on leakage and bursting rates at specific locations within the anastomosis.

## Results

**Fig. 7:**
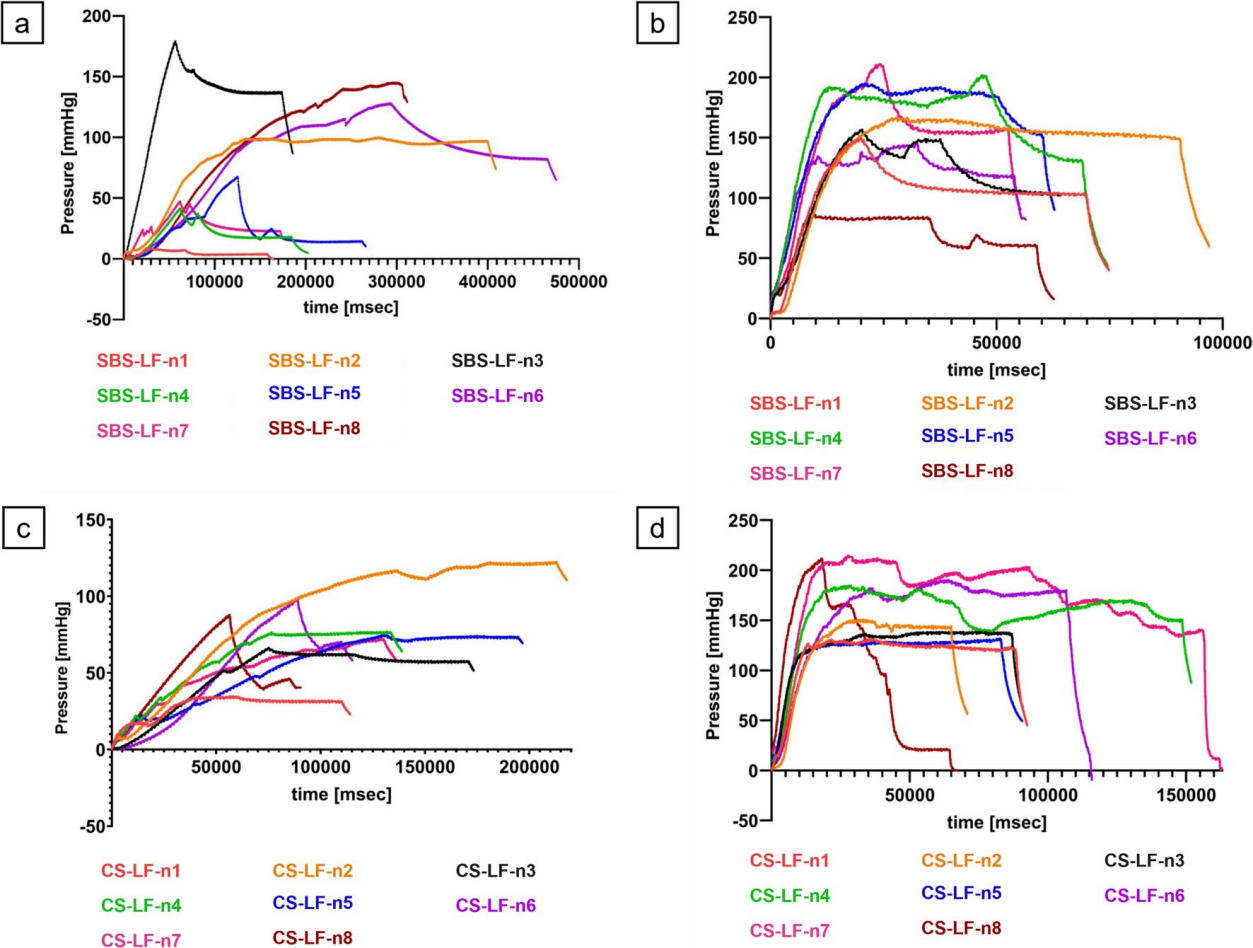
Pressure–time profiles of all anastomoses. a. SBS-LF anastomoses. b. SBS-HF anastomoses. c. CS-LF anastomoses. d. CS-HF anastomoses. mmHg = Millimeters of mercury; msec = Milliseconds

A total of 32 handsewn small intestinal end-to-end anastomoses were performed. Half of these anastomoses were constructed using the SBS technique (Supplementary Fig. 5), while the other half utilized the CS technique (Supplementary Fig. 6). Within each suture technique group, half of the anastomoses were tested in the LF model, while the remaining half were tested in the HF. Figure 7 illustrates the pressure–time profiles of the individual groups, whereby Fig. 7a presents the SBS-LF group, Fig. 7b the SBS-HF group, Fig. 7c the CS-LF group, and Fig. 7d the CS-HF group. Descriptive statistical analysis results for LP, BP, and time intervals can be found in Supplementary Tables 6–13. Interrelated analyses from experimental outcomes are presented in Supplementary Tables 10–13 and Supplementary Figs. 7–9.

### Quantitative analysis of anastomotic performance and time intervals: comparison within the experimental series

#### Leakage pressure analysis

**Fig. 8:**
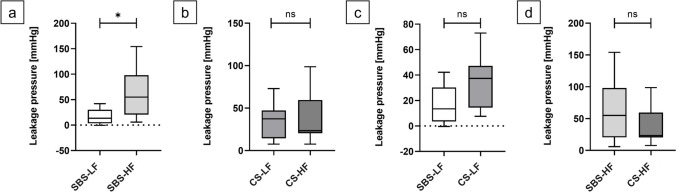
Leakage pressure (LP) comparison among experimental series. Box plots of anastomotic LP values (mmHg) comparing SBS-LF with SBS-HF anastomoses, CS-LF with CS-HF anastomoses, SBS-LF with CS-LF anastomoses and SBS-HF with CS-HF anastomoses. (**a**) SBS-HF anastomoses had a statistically significantly higher LP compared to SBS-LF anastomoses (*p* = 0.0281). (**b**) No significant difference in LP was seen between CS-LF and CS-HF (*p* = 0.9043); (**c**) SBS-LF and CS-LF (*p* = 0.0830) and (**d**) SBS-HF and CS-HF (*p* = 0.3823) anastomoses. Significance was assessed using Mann–Whitney U tests

For SBS-anastomoses, the HF model showed a statistically significantly higher LP compared to the LF model (*p* = 0.0281; exact 95.01% *CI* for the difference ranging from 4.200 to 81.50). (Fig. 8a) However, no statistically significant differences for LP were observed between the two flow models for CS-anastomoses (*p* = 0.9043; exact 95.01% *CI* of difference -23.8 to 24.80) (Fig. 8b). Additionally, there were no significant differences in LP between the two suture techniques, regardless of the flow model employed (SBS-LF vs. CS-LF anastomoses: *p* = 0.0830; exact 95.01% *CI* of difference -1.300 to 39.00 (Fig. 8c); SBS-HF and CS-HF anastomoses: *p* = 0.3823; exact 95.01% *CI* of difference -73.50 to 15.80 (Fig. 8d)).

#### Bursting pressure analysis

**Fig. 9:**
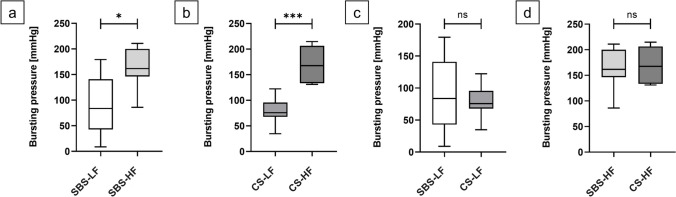
Bursting pressure (BP) comparison among experimental series. Box plots of anastomotic BP values (mmHg) comparing SBS-LF with SBS-HF anastomoses, CS-LF with CS-HF anastomoses, SBS-LF with CS-LF anastomoses and SBS-HF with CS-HF anastomoses. (**a**) SBS-HF anastomoses had a statistically significantly higher BP compared to SBS-LF anastomoses (*p* = 0.0115). (**b**) CS-HF anastomoses had a statistically significantly higher BP compared to CS-LF anastomoses (*p* = 0.0002). No significant difference in BP was seen between (**c**) SBS-LF and CS-LF (*p* = 0.7984) and (**d**) SBS-HF and CS-HF (*p* > 0.9999) anastomoses. Significance was assessed using Mann–Whitney U tests

For SBS-anastomoses (*p* = 0.0115; exact 95.01% *CI* for the difference ranging from 18.60 to 136.3) (Fig. 9a) and CS-anastomoses (*p* = 0.0002; exact 95.01% *CI* of difference of 56.60 to 124.0) (Fig. 9b), the HF model showed a statistically significant higher BP compared to the LF model.

However, no statistically significant differences for BP was observed between the two suture techniques, regardless of the flow model employed (SBS-LF vs. CS-LF anastomoses: *p* = 0.7984; exact 95.01% *CI* of difference -65.40 to 40.40 (Fig. 9c); SBS-HF and CS-HF anastomoses: *p* > 0.9999; exact 95.01% *CI* of difference -34.90 to 45.80 (Fig. 9d).

#### Time interval analysis

##### Time interval from start to leakage

For SBS-anastomoses (*p* = 0.0011; exact 95.01% *CI* for the difference ranging from -35,014 to -6537) (Supplementary Fig. 10.a) and CS-anastomoses (*p* = 0.0006; exact 95.01% *CI* of difference of -52,502 to -12,748) (Supplementary Fig. 10.b), LP was reached significantly faster in the HF model compared to the LF model. However, no statistically significant differences in time from start to LP was observed between the two suture techniques, regardless of the flow model employed (SBS-LF vs. CS-LF anastomoses: *p* = 0.6454; exact 95.01% *CI* of difference -11,620 to 27,900 (Supplementary Fig. 10.c); SBS-HF and CS-HF anastomoses: *p* = 0.7984; exact 95.01% *CI* of difference from -5519 to 1603 (Supplementary Fig. 10.d)).

##### Time interval from start to bursting

For SBS-anastomoses (*p* = 0.0011; exact 95.01% *CI* for the difference ranging from -251,503 to -29,704) (Supplementary Fig. 11.a) and CS-anastomoses (*p* = 0.0070 exact 95.01% *CI* of difference of -102,910 to -24,341) (Supplementary Fig. 11.b), BP was reached significantly faster in the HF model compared to the LF model. However, no statistically significant differences in time from start to BP was observed between the two suture techniques, regardless of the flow model employed (SBS-LF vs. CS-LF anastomoses: *p* = 0.7984; exact 95.01% *CI* of difference -148,167 to 69,700 (Supplementary Fig. 11.c); SBS-HF and CS-HF anastomoses: *p* = 0.0830; exact 95.01% *CI* of difference from -299.0 to 45,694 (Supplementary Fig. 11.d)).

##### Time interval from leakage to bursting

BP was reached significantly faster after LP for SBS-anastomoses in the HF model compared to the LF model (*p* = 0.0070; exact 95.01% *CI* for the difference ranging from -214,300 to -14,104) (Supplementary Fig. 12.a) and compared to CS-anastomoses in the HF model (*p* = 0.0379 exact 95.01% *CI* of difference of 141.0 to 50,402) (Supplementary Fig. 12.b). No significant differences in time from LP to BP were observed between the two flow models for CS-anastomoses (*p* = 0.0650, exact 95.01% *CI* of difference ranging from -76,674 to 3710) (Supplementary Fig. 12.c). Furthermore, no significant difference was observed between the LF model of both suture techniques (*p* = 0.9591; exact 95.01% *CI* of difference from -127,995 to 54,551 (Supplementary Fig. 12.d)).

### Descriptive analysis of leakage and bursting location

#### Leakage and bursting location analysis

**Fig. 10:**
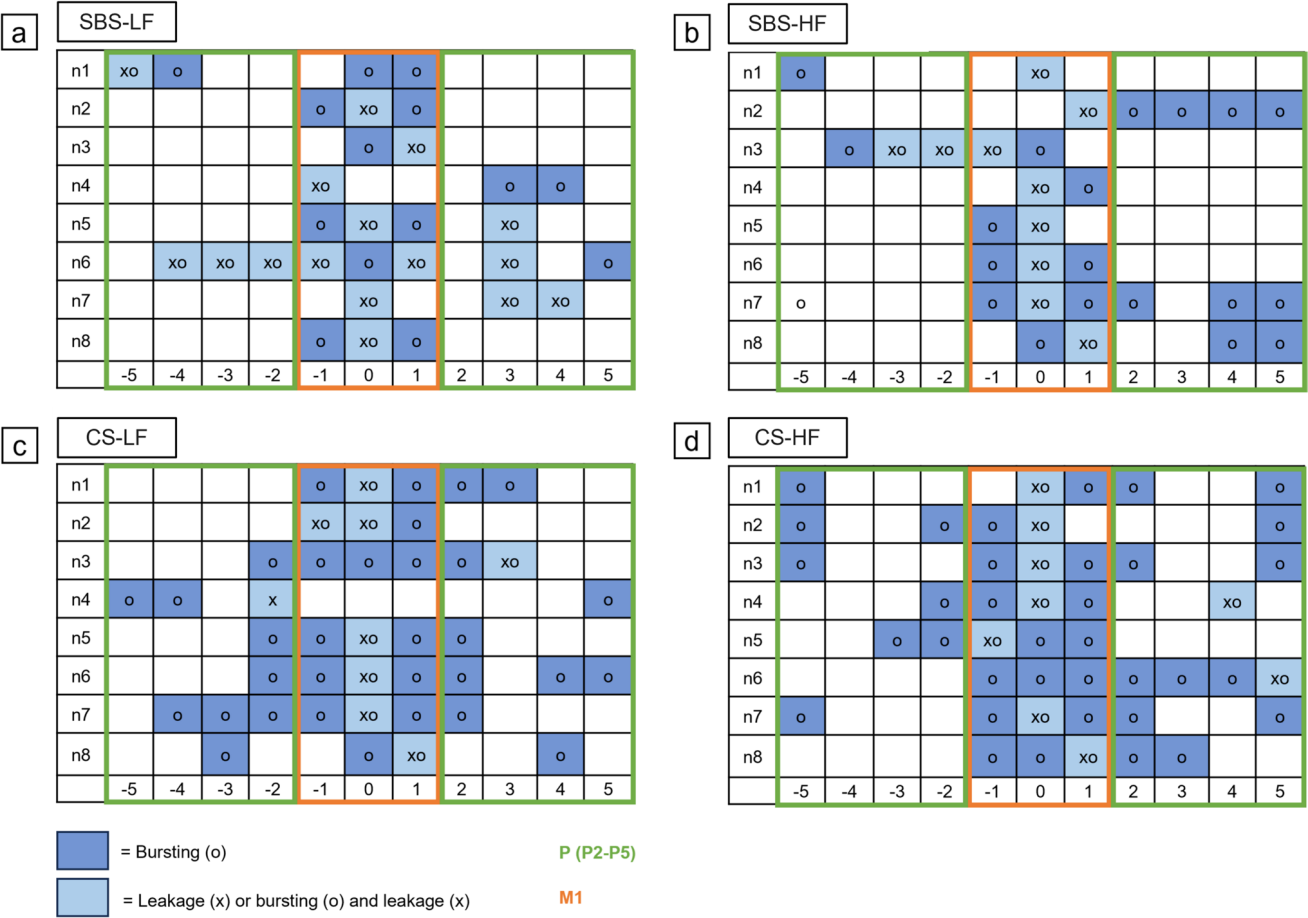
Location analysis: Spatial distribution of anastomotic leakage and bursting in the experimental series. The figure illustrates a linear grid, distinguishing between two zones: the mesenteric zone (between -1 and + 1, labeled as zone M1) and the peripheral zone (ranging from -2 to -5 and + 2 to + 5, labeled as zone P (P2-P5)). Leakage points are marked with "x" and bursting points with "o". (a) SBS-LF anastomoses. (b) SBS-HF anastomoses. (c) CS-LF anastomoses. (d) CS-HF anastomoses

For the SBS-LF and SBS-HF groups respectively, a total of eight (LP), 18 and 16 (BP) anastomotic locations with LP in the mesenteric zone were observed, while 16 (LP), six and eight (BP) anastomotic locations in the mesenteric zone did not exhibit leakage. In the peripheral zone, seven and two (LP), and twelve and 13 (BP) anastomotic locations displayed leakage, while 57 and 62 (LP), and 52 and 51 (BP) anastomotic locations remained free from leakage. For the CS-LF and CS-HF groups respectively, seven (LP), 20 and 22 (BP) and anastomotic locations exhibited leakage in the mesenteric zone, while 17 (LP), four and two (BP) anastomotic locations in the mesenteric zone did not show leakage. In the peripheral zone, two (LP), 20 and 22 (BP) anastomotic locations displayed leakage, while 62 (LP), 44 and 42 (BP) anastomotic locations remained free from leakage (Fig. 10).

#### Association between leakage and bursting locations

Among all cases of leakage observed in the study, with the exception of one, it was found that the anastomoses at the leakage location side also exhibited bursting. This consistent pattern suggests a strong association between leakage occurrence and the risk of bursting at the same location within the anastomosis.

### Leakage and bursting location analysis: comparison within and between the experimental series

No differences were observed in AL rates of areas within the mesenteric zone (Supplementary Fig. 13) and within the peripheral zone (Supplementary Fig. 14) among compared experimental series. Similarly, no differences were observed in BP rates of areas within the mesenteric zone (Supplementary Fig. 15) and within the peripheral zone (Supplementary Fig. 16) among compared experimental series.

#### Differences in anastomotic leakage rates between areas of the mesenteric and the peripheral zone

**Fig. 11:**
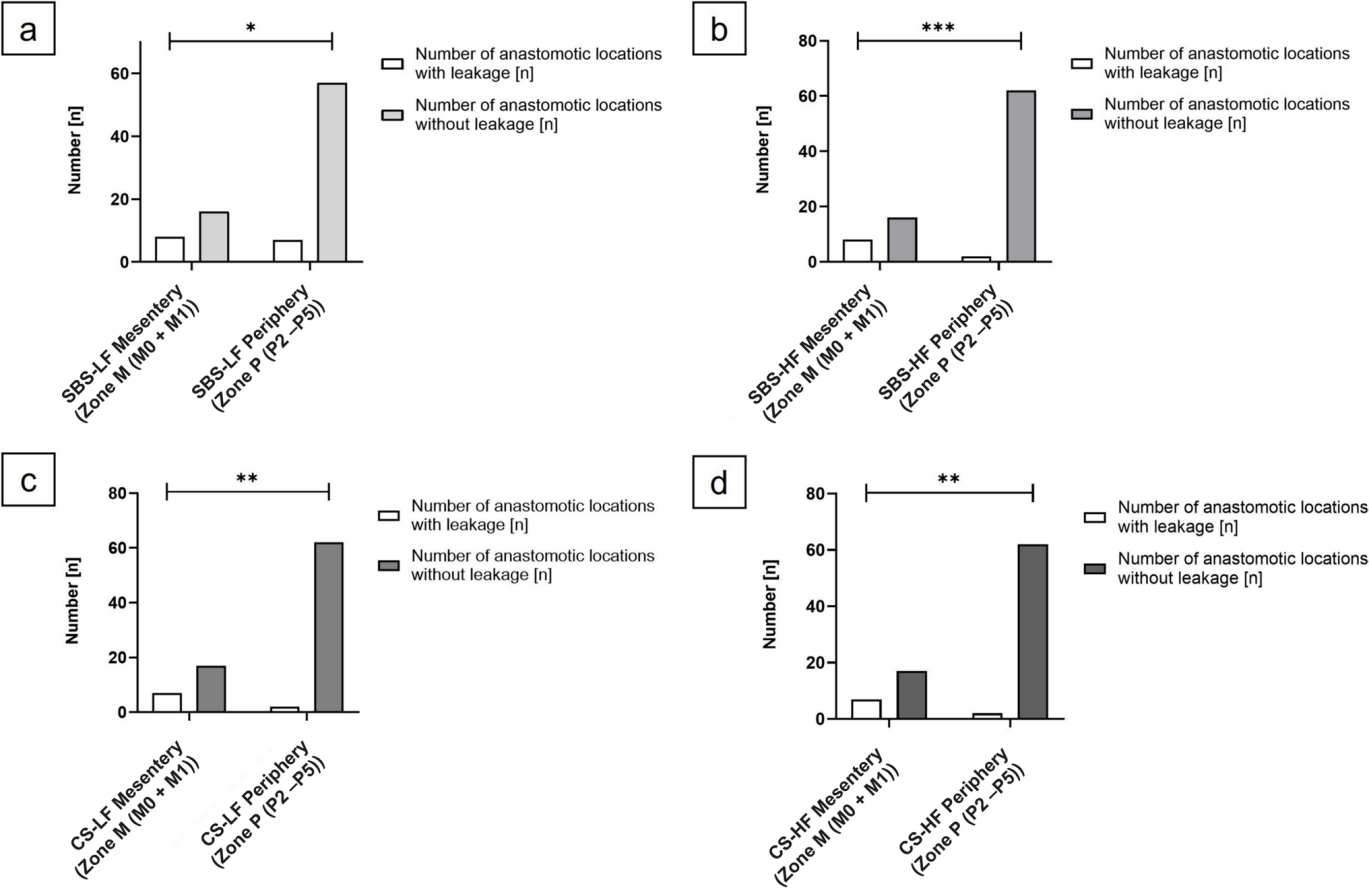
Differences in anastomotic leakage rates between the areas of the mesenteric and the peripheral zone. (a) SBS-LF anastomoses (*p* = 0.0230), (b) SBS-HF anastomoses (*p* = 0.0003), (c) CS-LF anastomoses (*p* = 0.0013), and (d) CS-HF anastomoses (*p* = 0.0013) exhibited a statistically significantly higher incidence of leakage at the mesenteric zone compared to the peripheral zone. Significance was assessed using Fisher’s exact test. *p < 0.05; ** *p* < 0.01; *** *p* =  < 0.001

The incidence of leakage in the SBS -LF anastomoses was significantly higher in the mesenteric zone compared to the peripheral zone (*p* = 0.0230; *OR*, 4.071). Similarly, SBS-HF anastomoses (*p* = 0.0003; *OR*, 15.50); CS-LF anastomoses (*p* = 0.0013; *OR*, 12.76), and CS-HF anastomoses (*p* = 0.0013; *OR*, 12.76) exhibited significantly higher incidences of leakage at the mesenteric zone compared to the peripheral zone (Fig. 11).

Therefore, irrespective of the anastomotic technique or flow rate model utilized, anastomoses exhibited a significantly higher incidence of leakage at the mesenteric entry side compared to other sections of the anastomosis.

#### Differences in anastomotic bursting rates between areas of the mesenteric and the peripheral zone

**Fig. 12:**
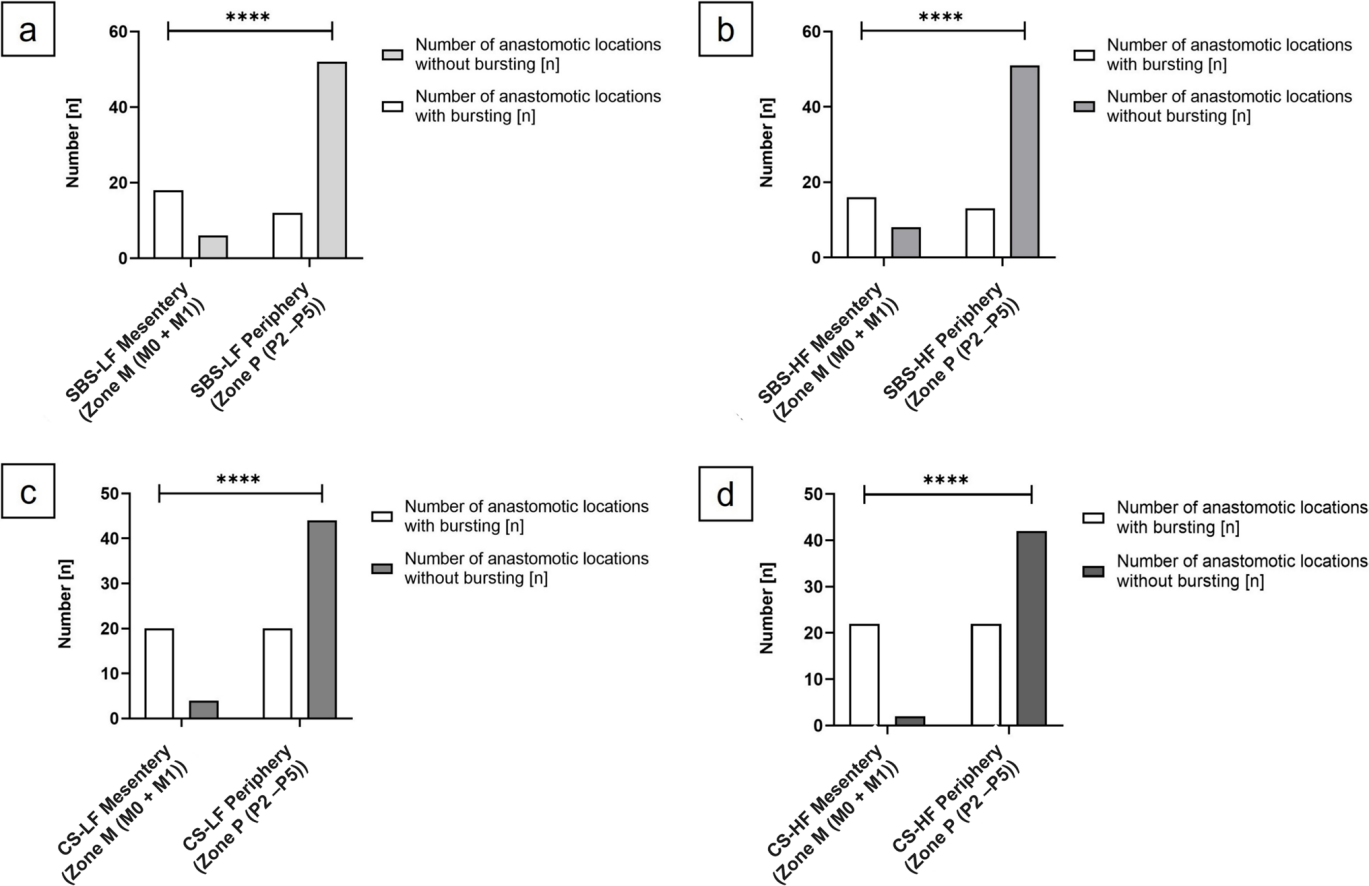
Differences in anastomotic bursting rates between the areas of the mesenteric and the peripheral zone. (a) SBS-LF anastomoses (*p* < 0.0001), (b) SBS-HF anastomoses (*p* < 0.0001), (c) CS-LF anastomoses (*p* < 0.0001), and (d) CS-HF anastomoses (*p* < 0.0001) exhibited a statistically significantly higher incidence of bursting at the mesenteric zone compared to the peripheral zone. Significance was assessed using Fisher’s exact test. *p < 0.05; ** *p* < 0.01; *** *p* =  < 0.001; **** *p* < 0.0001

Significantly higher incidences of bursting in the SBS-LF anastomoses were observed in the mesenteric zone compared to the peripheral zone (*p* < 0.0001; *OR*, 13.00). Similarly, SBS-HF anastomoses zone (*p* < 0.0001; *OR*, 7.846), CS-LF anastomoses (*p* < 0.0001; *OR*, 11.00), and CS-HF anastomoses (*p* < 0.0001; *OR*, 21.00) exhibited a significantly higher incidence of bursting at the mesenteric zone compared to the peripheral (Fig. 12).

Therefore, irrespective of the experimental series or anastomotic technique used, anastomoses exhibited a significantly higher incidence of bursting at the mesenteric entry side compared to other sections of the anastomosis.

## Discussion

The herein used *ex-vivo* model allows for comparable, reproducible and user-independent investigation of anastomotic biomechanics under controlled conditions. It is important to note that the goal was not to precisely replicate *in-vivo* intestinal stress but rather to induce stress on the tissue wall in a manner reflective of observed human stress situations. This approach allows analysis of biomechanical behavior, specifically stretching and stiffness, and offers the advantage of identifying technical weak points of gastrointestinal anastomoses. Surgeons can utilize this information to improve their techniques and minimize technical-associated AL.

Results from the experiment unveiled significant differences in LP, BP and time intervals, influenced by the chosen flow models (Figs. 8 and 9; Supplementary Figs. 7–12). These variances likely stem from time-dependent stress–strain reactions inherent in the viscoelastic properties of biological tissues. Rapid influx of fluid leads to an abrupt pressure surge due to immediate elastic response, but viscous behavior causes continuous stretching over time until the tissue eventually bursts. In contrast, under slow loading conditions, the viscous tissue element has more time to react, resulting in a gradual stress–strain response. When stress is removed, faster relaxation occurs. Consequently, with a lower rate of intraluminal fluid influx, the tissue experiences a gradual pressure increase, matching its viscous response to the pace of deformation. This leads to the attainment of a lower final pressure before tissue bursting [24–27, 50–52]. Importantly, the choice of suture technique appeared to have no considerable impact on either LP or BP, indicating minimal influence on the stability and pressure resistance of the end-to-end anastomoses. The fact that these results can be detected by the *ex-vivo* model further strengthens its relevance in investigating anastomotic biomechanics.

One of the central findings emphasize the association between leakage and bursting and the specific location within the anastomosis, particularly the insertion site of the mesentery. This supports the assumption that the insertion site of the mesentery represents a vulnerable point where AL can develop. The insertion site of the mesentery serves as a potential weak spot due to its role as a transition area between the mobile and fixed parts of the intestine. Here, the mesentery, responsible for blood supply and nerve innervation, attaches to the intestinal wall, creating a point of stress concentration that increases the susceptibility to mechanical failure or disruption [53]. Anastomoses performed at the insertion site of the mesentery during surgical procedures compound the risk as they weaken the tissue and elevate the likelihood of complications such as AL. However, factors such as suture quality, tissue healing response, and mechanical stress also contribute to the risk of leakage in this specific anatomical location [54–58].

Given the clinical significance of anastomotic complications, careful evaluation and management of the insertion site of the mesentery during surgical procedures are crucial. Surgeons should exercise caution and employ appropriate techniques to ensure secure and reliable anastomoses at this critical location. Exploring advancements in surgical techniques, including reinforcement methods or innovative approaches to strengthen the anastomotic site, is essential to mitigate the risk of complications and improve patient outcomes. By gaining a better understanding of potential weak spots in anastomoses, such as the insertion site of the mesentery, surgeons can make informed decisions and implement strategies to minimize the occurrence of AL. Collaborative efforts between surgeons and researchers, along with future research endeavors, are warranted to refine surgical techniques, develop novel interventions, and enhance patient safety in the context of anastomotic procedures.

The inherent limitations of the model align with challenges commonly observed in *ex-vivo* models, as they face difficulties in reproducing the complex physiological conditions present in *in-vivo* settings. Variables such as blood flow, tissue perfusion, and dynamic physiological forces are inadequately simulated. The *ex-vivo* model was intentionally designed to focus on investigating the biomechanical properties of various anastomotic techniques and the effects of different pressure conditions. While acknowledging the inability to fully replicate *in-vivo* complexity, deliberate simplifications and controlled conditions allow for isolating and scrutinizing specific biomechanical aspects. This focus aligns with the rationale that a comprehensive understanding of these mechanical factors is crucial for refining surgical techniques and optimizing outcomes. While this model may not capture the full spectrum of biological complexities, its intentional design provides a valuable niche for investigating biomechanical dimensions critical to anastomotic procedures. Further research can build upon these findings to enhance our understanding of broader biological healing processes in gastrointestinal surgeries.

Another limitation important to note is the variability among the samples. The test setup aimed to objectively quantify *ex-vivo* AL and bursting detection by standardizing parameters essential biomechanical measurements, such as temperature, pH or pressure differences. It is crucial to recognize that the experimental model is intentionally crafted to accommodate inherent variations, especially within an *ex-vivo* context where tissue-related factors and surgical techniques introduce an anticipated level of variability. Despite this acknowledged variation, the model has demonstrated its ability to discern significant differences, such as distinguishing between LF and HF models. Furthermore, the potential impact of sample size on variation is recognized and increasing it could additionally alleviate this concern. These considerations underscore the robustness of the model in capturing and distinguishing pertinent biomechanical properties.

Finally, a standardization and automation of the *ex-vivo* model for suture evaluation represent a commendable initiative with the potential to pave the way for future regulatory frameworks governing the authorization of new stapling systems. By establishing a uniform and automated testing protocol, the reliability and reproducibility of suture evaluations can be significantly enhanced. This not only ensures consistency in assessing the performance of various stapling systems but also provides a basis for developing robust regulatory guidelines. Such guidelines, rooted in standardized testing methodologies, can contribute to the systematic evaluation of new stapling systems, fostering innovation, safety, and efficacy in surgical practices. The integration of these advancements into regulatory frameworks may prove instrumental in shaping the landscape of stapling system authorization, promoting advancements in surgical technology, and enhancing patient care.

In conclusion, this study provides a valuable contribution to the understanding of gastrointestinal anastomoses’ biomechanics through an application of an operator-independent *ex-vivo* model generating comparable and reproducible results. The experimental results revealed significant differences in anastomoses’ LP, BP, and time intervals based on flow models, attributed to their inherent biomechanical properties as biologic tissue. The choice of suture technique did not significantly affect LP and BP, suggesting that it may not have a substantial impact on anastomotic stability and pressure resistance. Most interestingly, the observed higher leakage and bursting rates at the insertion site of the mesentery suggest that this site displays specific biomechanical properties, rendering it a potential weak spot for anastomosis, and making it more prone to AL development. Surgical techniques and devices should adapt to this potential weak spot to minimize the risk of mechanically induced AL.

### Supplementary Information

Below is the link to the electronic supplementary material.

Supplementary file1 (DOCX 8310 KB)

## Data Availability

The authors confirm that the data supporting the findings of this study are available within the article and its supplementary materials.
