# Supplementary material for: The mesenteric entry site as a potential weak point in gastrointestinal anastomoses – findings from an ex-vivo biomechanical analysis
Source: Langenbecks Arch Surg. 2024 Apr 13;409(1):124. doi: 10.1007/s00423-024-03318-8 (PMC11016002; doi:10.1007/s00423-024-03318-8)
Supplement: Supplementary file 1 — Supplementary file1 (DOCX 8310 KB) [file 423_2024_3318_MOESM1_ESM.docx]

1. **Supplementary Figures**

**1.1 Supplementary Figure 1**

**
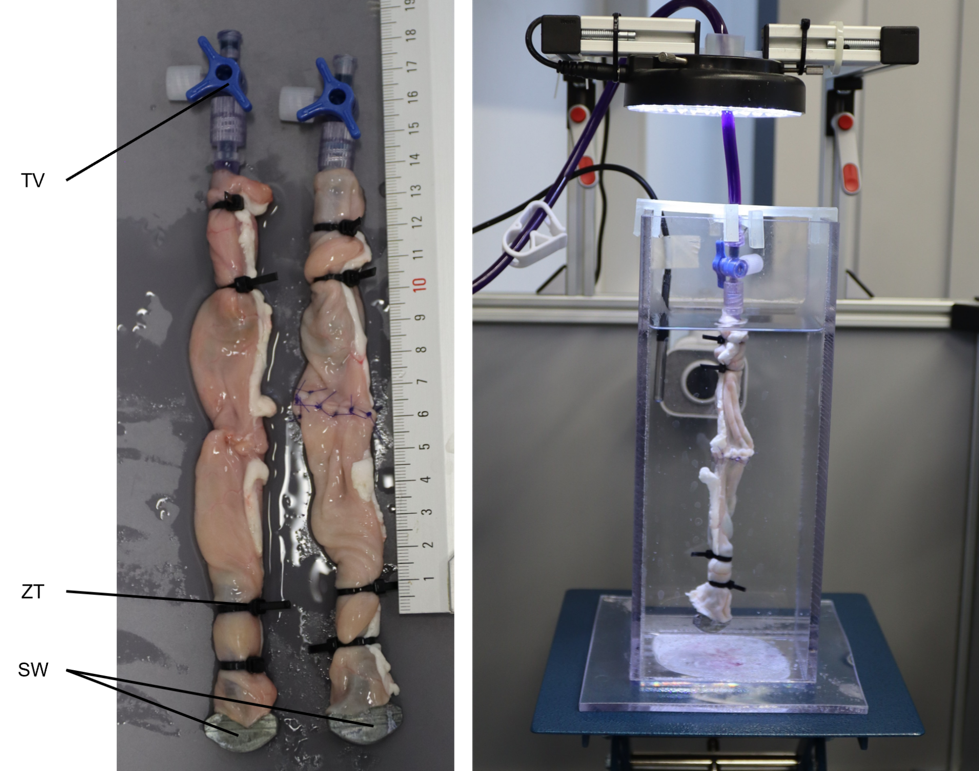
**

**Supplementary Fig. 1 Anastomotic unit.** SW = Stainless steel screw; TV = Three-way-valve; ZT = Zip ties

**1.2. Supplementary Figure 2**

**
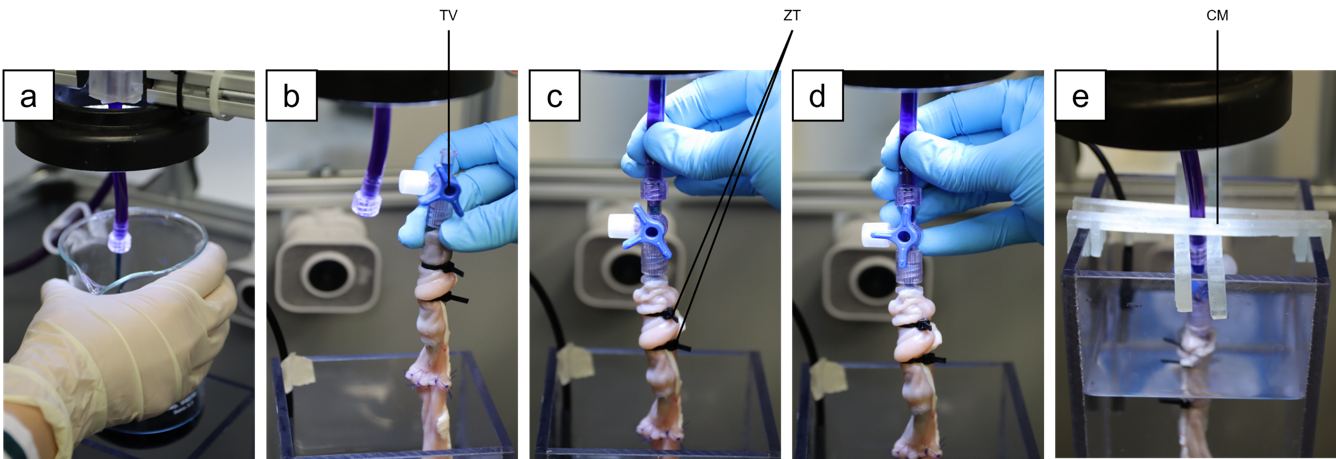
**

**Supplementary Fig. 2 Connection of intestinal anastomosis to the mechanical drive unit.** The figure illustrates the steps involved in connecting the intestinal anastomosis to the mechanical drive unit for experimental testing: (a) The flushing process with methyl green-colored phosphate buffered saline solution effectively eliminates any trapped air. (b) The flushed and closed three-way-valve is securely connected to the intestinal anastomosis using zip ties, ensuring a tight seal. (c) The flushed and closed three-way-valve is then connected to the flushed and airless tube from the mechanical drive unit. (d) The flushed three-way-valve is subsequently opened, allowing controlled fluid flow during the experiment. (e) Finally, the intestinal anastomosis is carefully positioned in the testing chamber, which is filled with a phosphate buffered saline solution maintained at a temperature range of 35 – 39 °C, creating a physiological environment for testing. CM = Custom made 3D-printes stabilization brackets; TV = Three-way-valve; ZT = Zip ties

**1.3 Supplementary Figure 3**

**
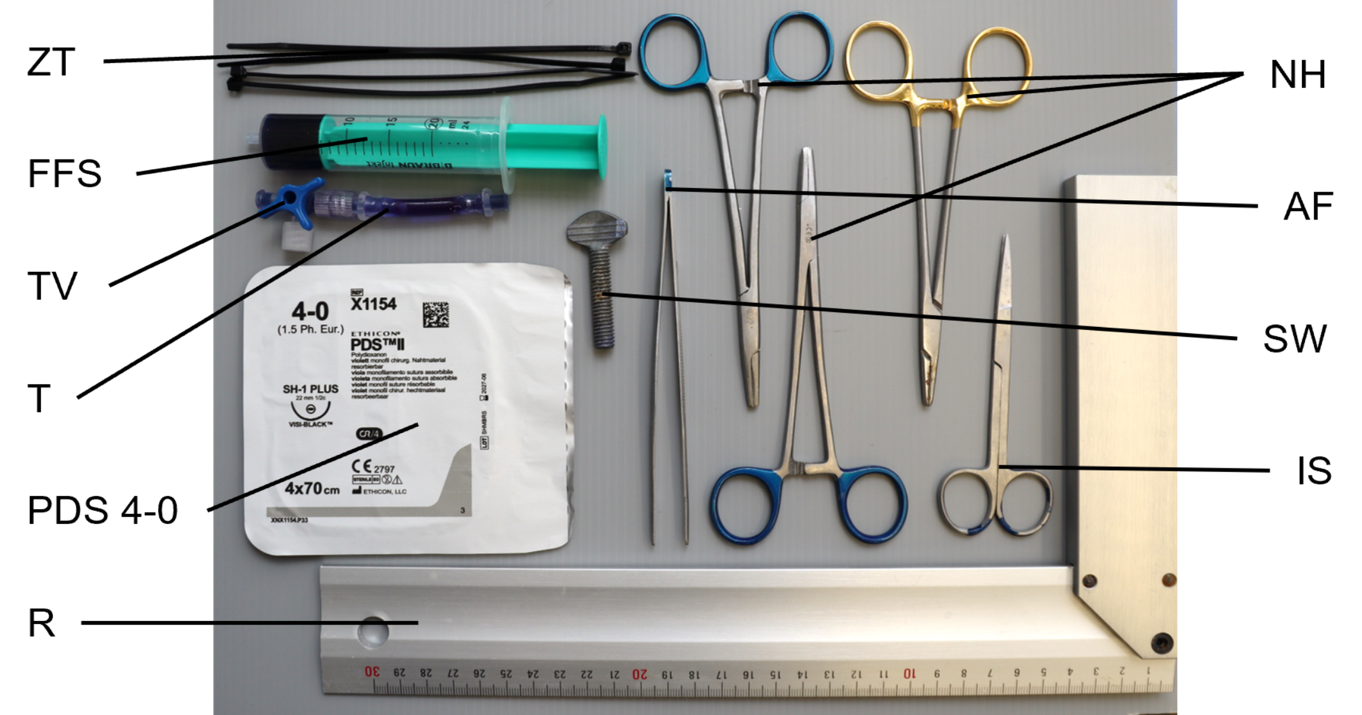
**

**Supplementary Fig. 3 Instruments and materials for creating the anastomotic unit.** AF = Anatomical forceps; FFS = Fluid-filled syringe; IS = Iris scissors; NH = Needle holder; R = Ruler; SW = Stainless steel screw; T = Tube; TV = Three-way-valve; ZT = Zip ties

**1.4 Supplementary Figure 4**

**
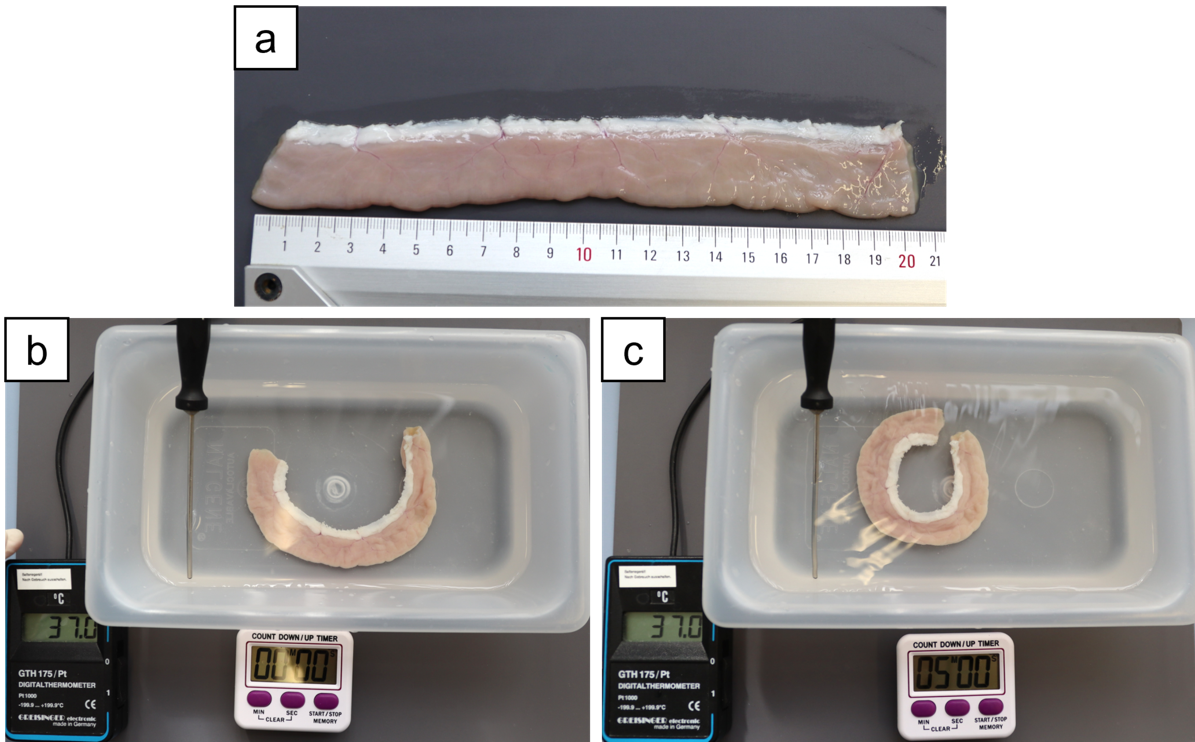
**

**Supplementary Fig. 4 Preparation and rehydration of porcine small intestine.** (a) The porcine small intestines were meticulously dissected into 20 cm long segments before their utilization in the experiment. (b) Prior to performing the intestinal anastomosis, the tissues underwent a rehydration process and were heated to 37 °C. (c) The porcine small intestines used in the experiments were pre-rehydrated for at least 5 minutes in 37 °C phosphate buffered saline solution both before and after performing the intestinal anastomosis

**1.5 Supplementary Figure 5**

**
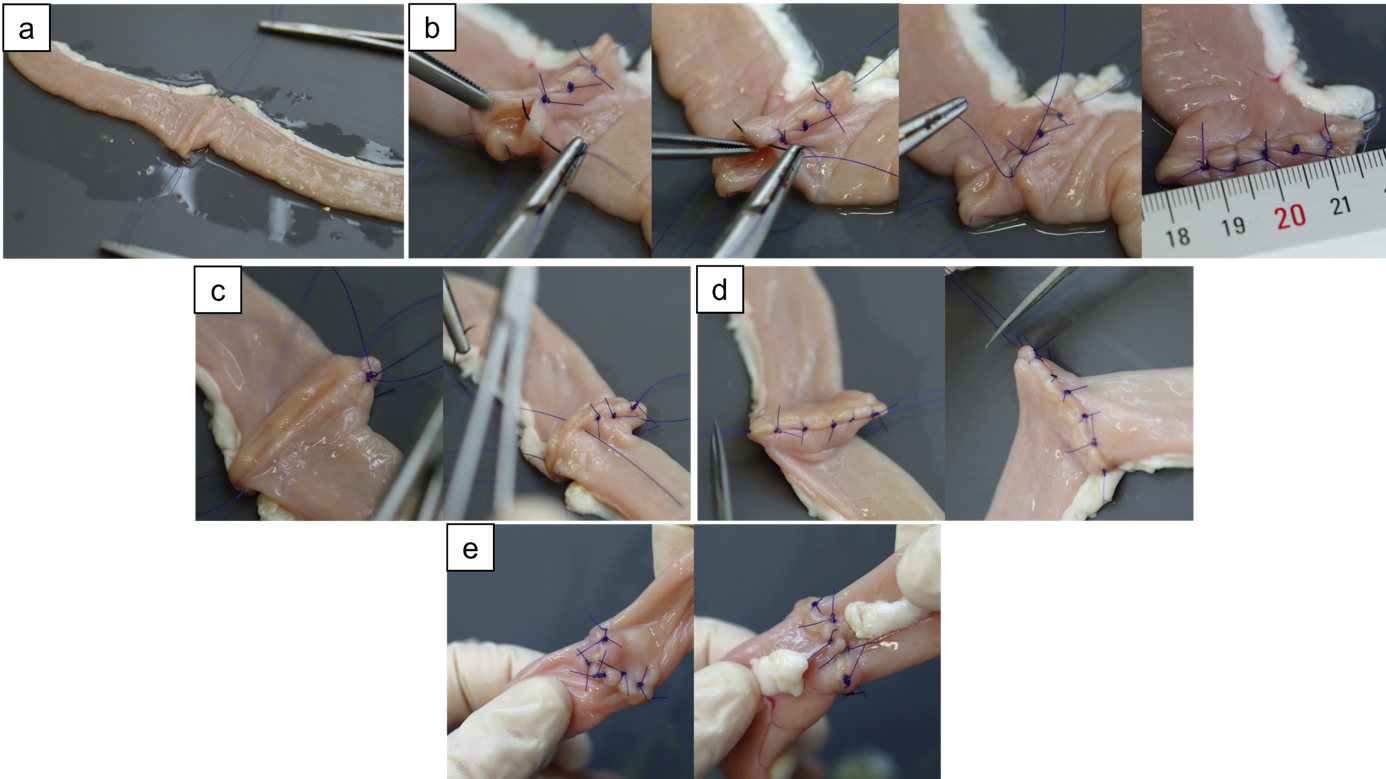
**

**Supplementary Fig. 5 Handsewn sufficient small intestinal end-to-end anastomosis using the interrupted suture technique.** (a) A seromuscular stay suture was placed 5 mm away from the incision site at the proximal and distal ends of the intestinal segments. (b) Interrupted sutures were placed between the two stay sutures, maintaining a 5 mm distance to the intestinal margin, with a stitch distance of 5 mm. (c) After securely closing the anterior site of the intestinal segments, the intestinal segment was rotated by 180° to visualize the unsutured posterior segment, and the anastomosis was closed from the antimesenteric border to the mesenteric border. (d) Finally, both stay sutures were tied. (e) Completed handsewn sufficient small intestinal end-to-end anastomosis using interrupted suture technique, anterior and posterior views. 4-0 Polydioxanone (PDS) was utilized as suture material

**1.6 Supplementary Figure 6**

**
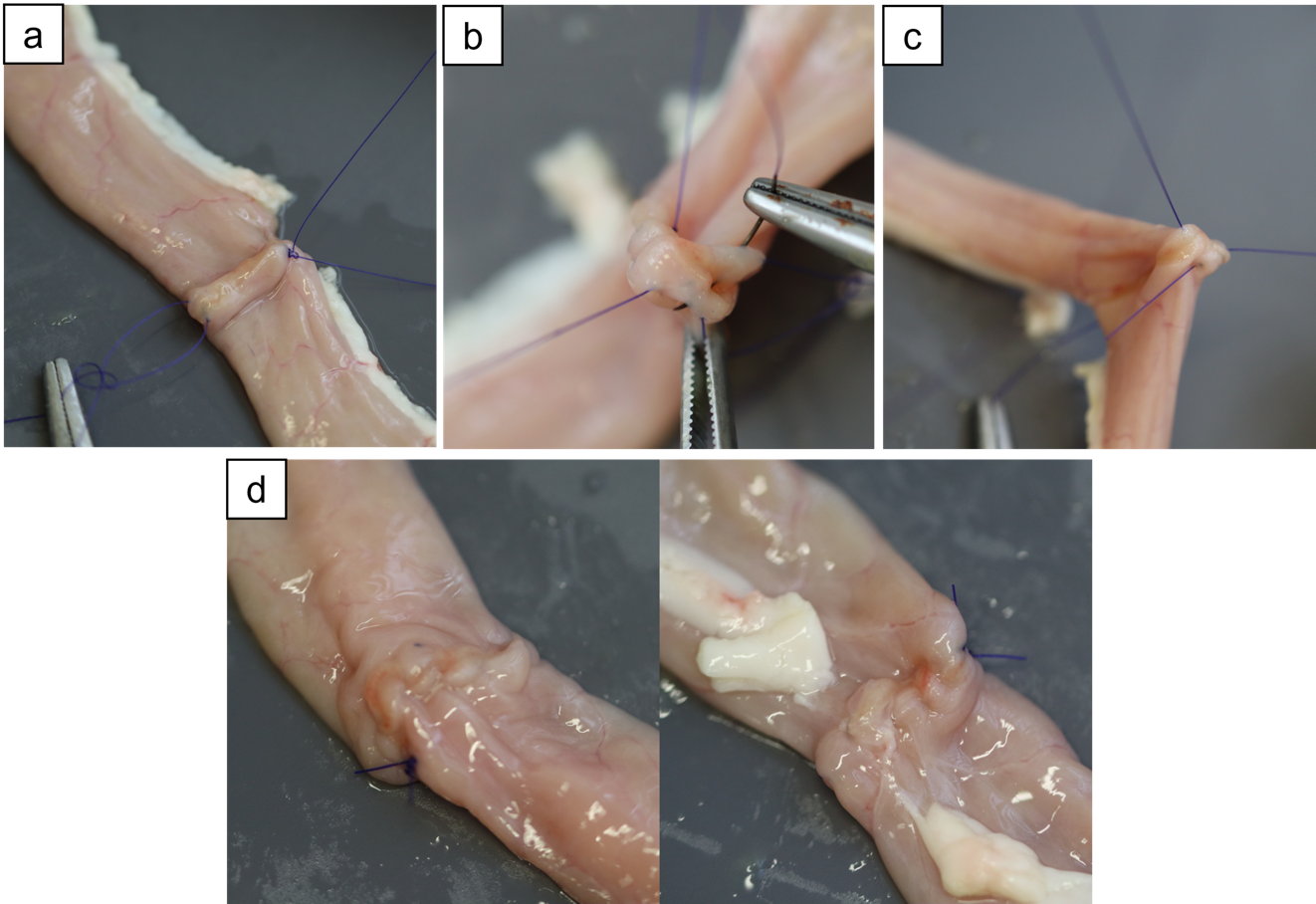
**

**Supplementary Fig. 6 Handsewn sufficient small intestinal end-to-end anastomosis using simple continuous suture technique.** (a) The first seromuscular stitch was placed 5 mm from the incision site at the mesenteric border, ensuring proper adaptation of both segment endings. A stay suture was positioned at the antimesenteric border, also 5 mm from the incision line. (b) Using the continuous seromuscular technique, the intestinal segments were sutured from the mesenteric border to the antimesenteric border. (c) After closing the anterior site, the intestinal segment was rotated by 180° to visualize the unsutured posterior intestinal segment, and the anastomosis was closed from the antimesenteric to the mesenteric border. (d) Finally, both suture ends were securely tied together with 6 knots. 4-0 Polydioxanone (PDS) was utilized as suture material

**1.7 Supplementary Figure 7**


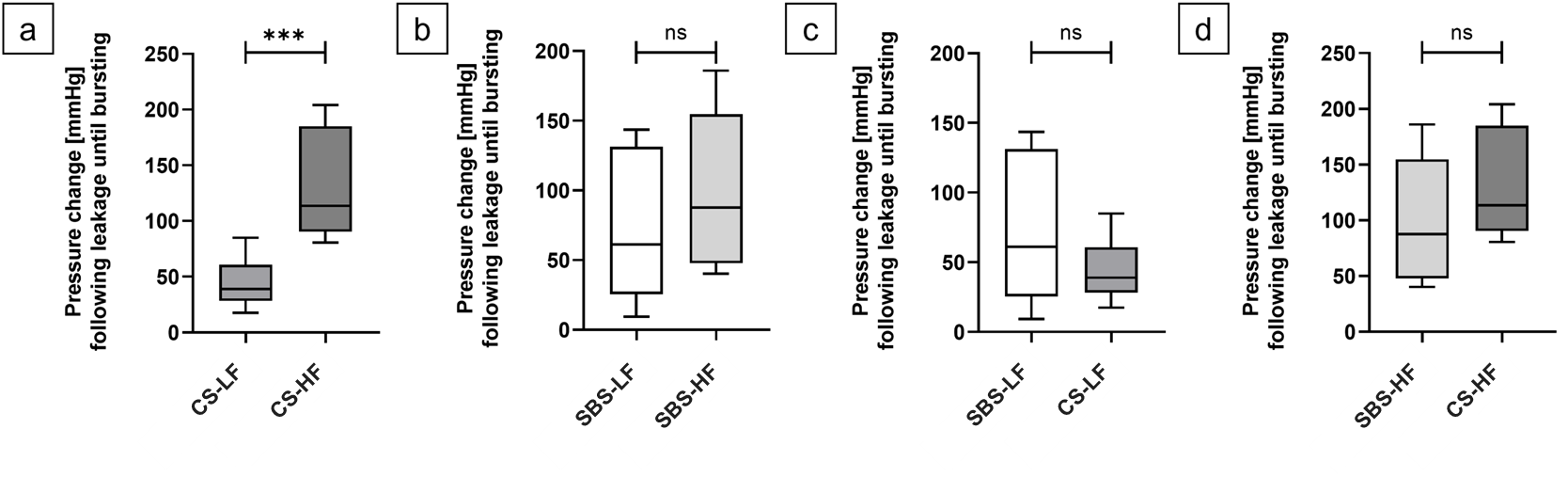


**Supplementary Fig. 7 Comparison of the proportion of bursting pressure (BP) at leakage pressure (LP) (in mmHg) between the experimental series. Box plots illustrating the pressure change (in mmHg) following LP until BP comparing CS-LF with CS-HF anastomoses, SBS-LF with SBS-HF anastomoses, SBS-LF with CS-LF anastomoses and SBS-HF with CS-HF anastomoses.** (a) After reaching LP, CS-HF anastomoses had a statistically significantly higher increase in intraluminal pressure until BP compared to CS-LF anastomoses (*p* = 0.0003). No significant difference in intraluminal pressure increase after LP until BP was observed between (b) SBS-LF and SBS-HF anastomoses (*p* = 0.2786), (c) SBS-LF and CS-LF anastomoses (*p* = 0.4418) and (d) SBS-HF and CS-HF anastomoses (*p* = 0.1605). Significance was assessed using Mann-Whitney U tests

**1.8 Supplementary Figure 8**


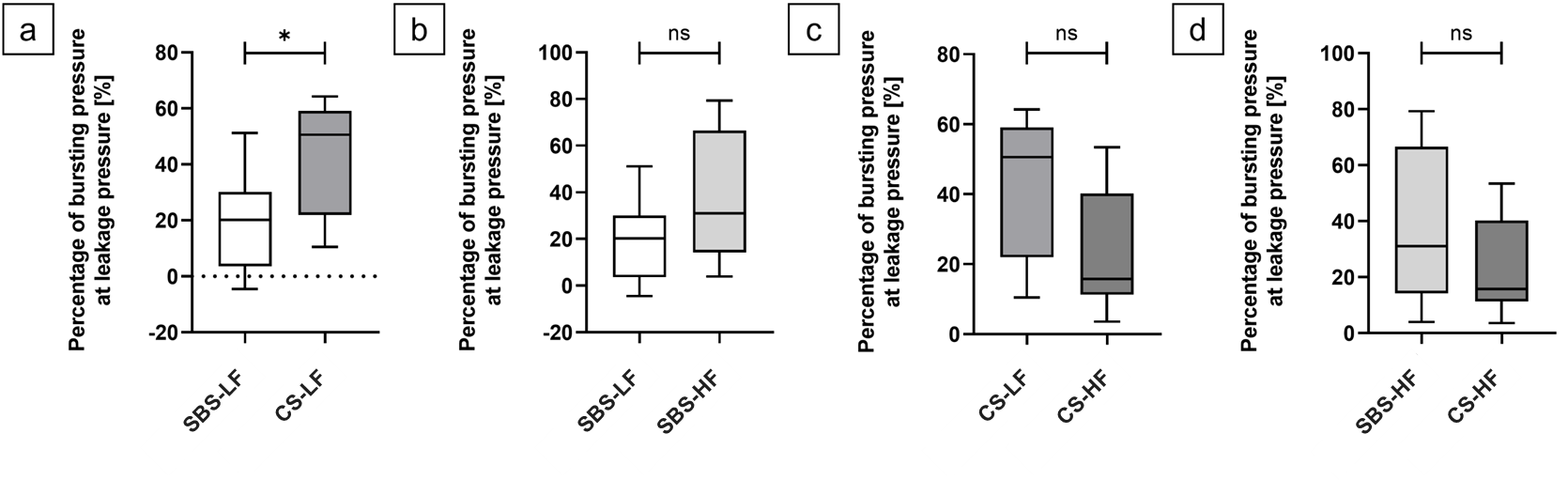


**Supplementary Fig. 8 Comparison of the percentage of bursting pressure (BP) at leakage pressure (LP) (in %) between the experimental series. Box plots illustrating the percentage of BP at LP (in %) comparing SBS-LF with CS-LF anastomoses, SBS-LF with SBS-HF anastomoses, CS-LF with CS-HF anastomoses and SBS-HF with CS-HF anastomoses.** (a) CS-LF anastomoses showed a statistically significantly relatively higher increase in intraluminal pressure after LP before reaching the point of bursting compared to SBS-LF anastomose (*p* = 0.0499). No significant difference in the percentage of BP at LP were seen between (b) SBS-LF and SBS-HF anastomoses (*p* = 0.1691), (c) CS-LF and CS-HF anastomoses (*p* = 0.0830) and (d) SBS-HF and CS-HF anastomoses (*p* = 0.1949). Significance was assessed using Mann-Whitney U tests

**1.9 Supplementary Figure 9**

**
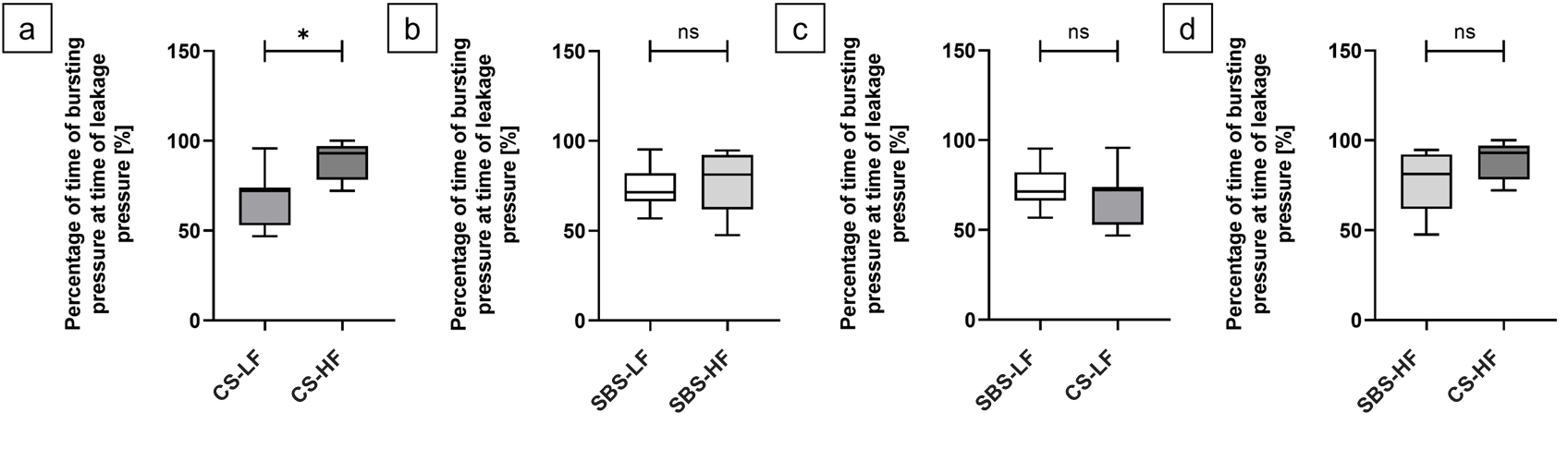
**

**Supplementary Fig. 9 Comparison of the time of leakage occurrence relative to the bursting time (in %) between the experimental series. Box plots illustrating the percentage of time of BP at the time of LP (in %) comparing CS-LF with CS-HF anastomoses, SBS-LF with SBS-HF anastomoses, SBS-LF with CS-LF anastomoses and SBS-HF with CS-HF anastomoses.** (a) CS-HF anastomoses statistically significantly reached BP faster after LP compared to CS-LF (*p* = 0.0148). No significant difference in the percentage of time of BP at the time of LP were seen between (b) SBS-LF and SBS-HF anastomoses (*p* = 0.5737), (c) SBS-LF and CS-LF anastomoses (*p* = 0.6454) and (d) SBS-HF and CS-HF anastomoses (*p* = 0.1049). Significance was assessed using Mann-Whitney U tests

**1.10 Supplementary Figure 10**


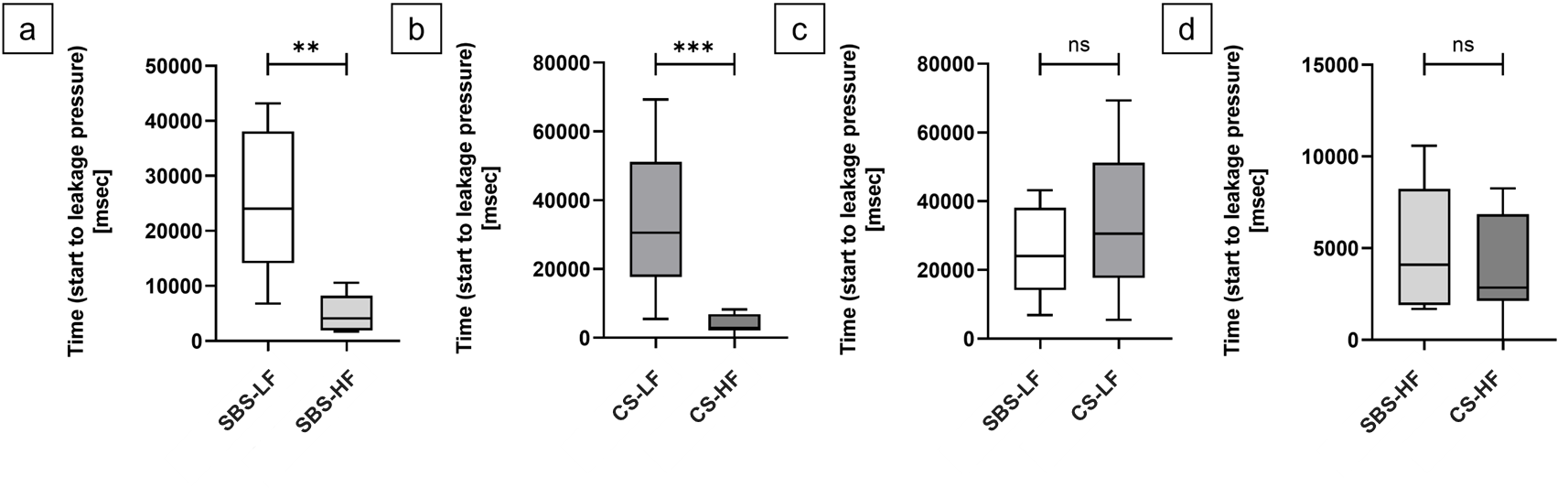


**Supplementary Fig. 10 Comparison of time interval assessment for attainment of leakage pressure (LP) in the experimental series. Box plots illustrating the time interval (in msec) for achieving LP comparing SBS-LF with SBS-HF anastomoses, CS-LF with CS-HF anastomoses, SBS-LF with CS-LF anastomoses and SBS-HF with CS-HF anastomoses.** (a) SBS-HF anastomoses had a statistically significantly shorter duration to reach LP compared to SBS-LF anastomoses (*p* = 0.0011). (b) CS-HF anastomoses had a statistically significantly shorter duration to reach LP compared to CS-LF anastomoses (*p* = 0.0006). No significant difference in time required to reach LP was seen between (c) SBS-LF and CS-LF (*p* = 0.6454) and (d) SBS-HF and CS-HF (*p* = 0.7984) anastomoses. Significance was assessed using Mann-Whitney U tests

**1.11 Supplementary Figure 11**


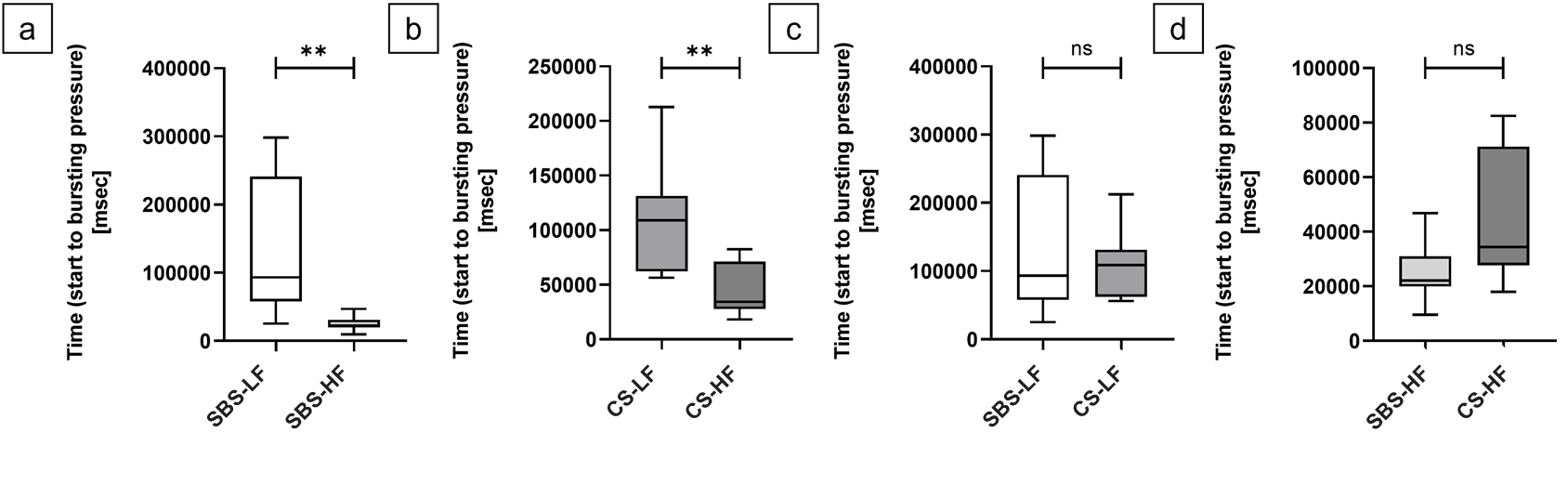


**Supplementary Fig. 11. Comparison of time interval assessment for attainment of bursting pressure (BP) in the experimental series. Box plots illustrating the time interval (in msec) for achieving BP comparing SBS-LF with SBS-HF anastomoses, CS-LF with CS-HF anastomoses, SBS-LF with CS-LF anastomoses and SBS-HF with CS-HF anastomoses.** (a) SBS-HF anastomoses attained statistically significantly faster BP compared to SBS-LF anastomoses (*p* = 0.0011). (b) CS-HF anastomoses attained statistically significantly faster BP compared to CS-LF anastomoses (*p* = 0.0070). No significant difference in time required to reach BP was seen between (c) SBS-LF and CS-LF (*p* = 0.7984) and (d) SBS-HF and CS-HF (*p* = 0.0830) anastomoses. Significance was assessed using Mann-Whitney U tests

**1.12 Supplementary Figure 12**


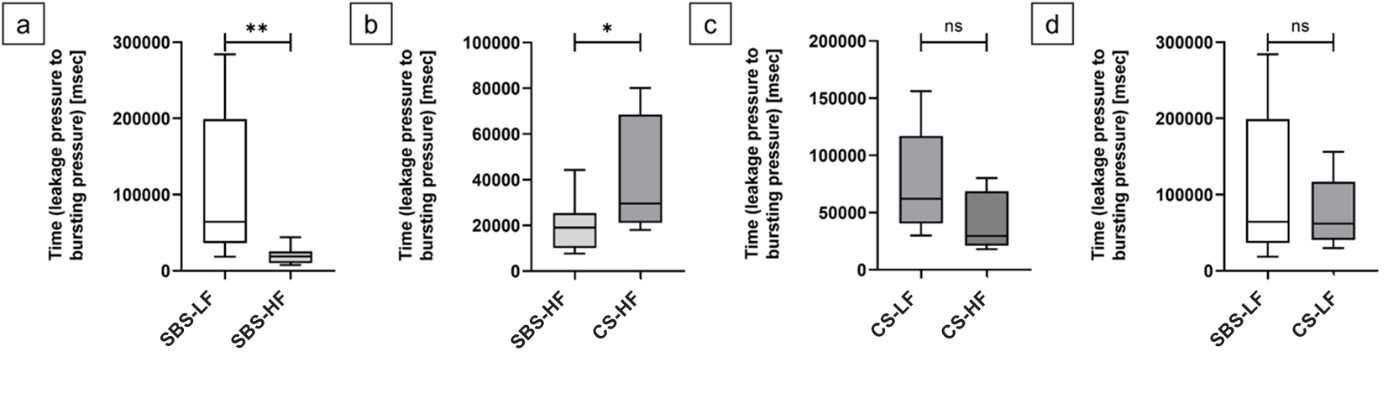


**Supplementary Fig. 12 Comparison of time interval assessment for attainment of bursting pressure (BP) in the experimental series. Box plots illustrating the time interval (in msec) for achieving BP after LP comparing SBS-LF with SBS-HF anastomoses, SBS-HF with CS-HF anastomoses, CS-LF with CS-HF anastomoses and SBS-LF with CS-LF anastomoses.** (a) SBS-HF anastomoses attained statistically significantly faster BP after LP compared to SBS-LF anastomoses (*p* = 0.0070). (b) SBS-HF anastomoses attained statistically significantly faster BP after LP compared to CS-HF anastomoses (*p* = 0.0379). (c) No significant difference in time required to reach BP after LP was seen between (c) CS-LF and CS-HF (*p* = 0.0650) and (d) SBS-LF and CS-LF (*p* = 0.9591) anastomoses. Significance was assessed using Mann-Whitney U tests

**1.13 Supplementary Figure 13**


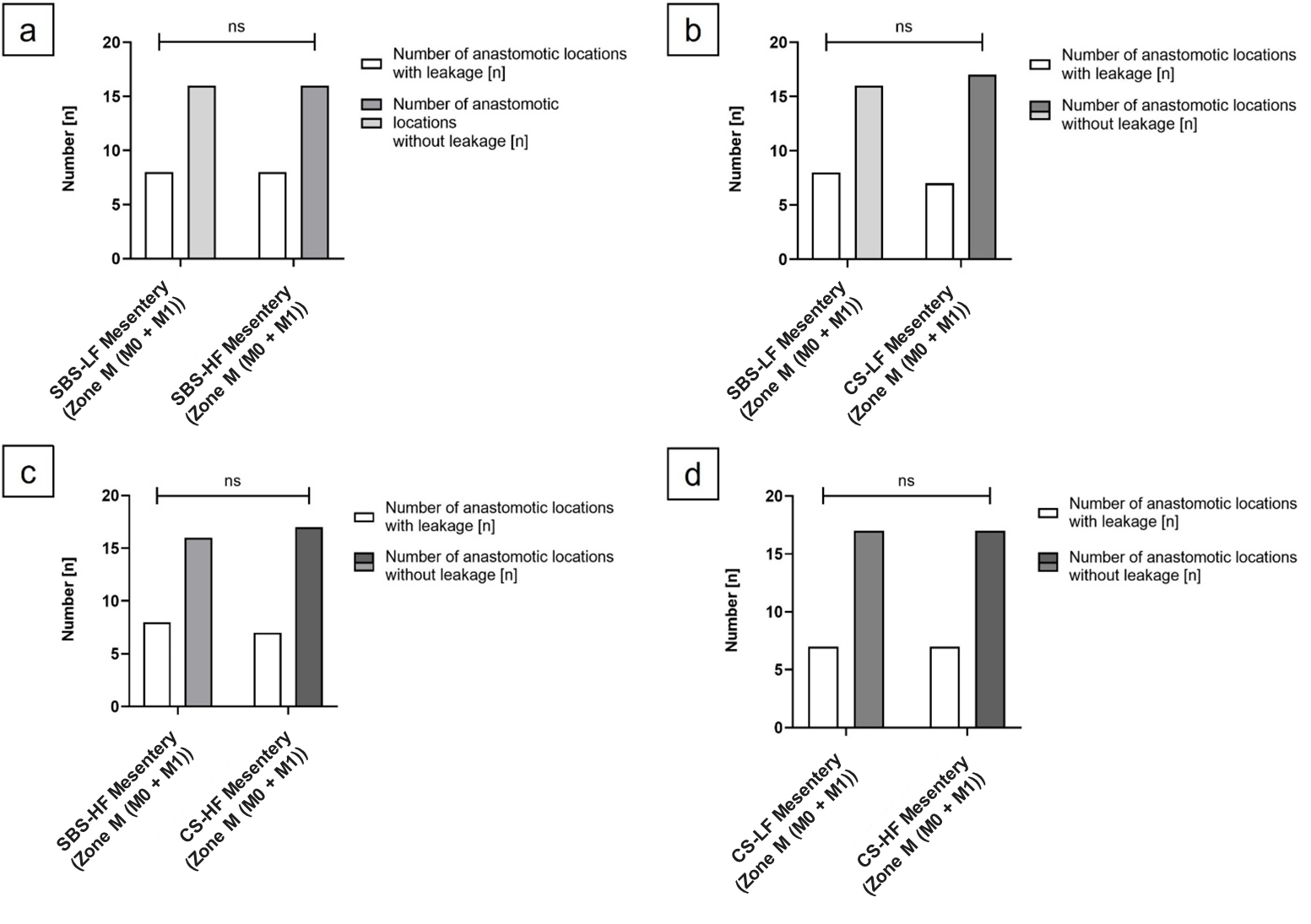


**Supplementary Fig. 13 Differences in anastomotic leakage rates of areas within the mesenteric zone among compared experimental series.** There were no statistically significant differences in the incidence of leakage at the mesenteric zone between (a) SBS-LF and SBS-HF anastomoses (p > 0.9999), (b) SBS-LF and CS-LF anastomoses ($p$ $> 0.9999$), (c) SBS-HF and CS-HF anastomoses ($p$ $> 0.9999$), and (d) CS-LF and CS-HF anastomoses ($p$ $> 0.9999$). Significance was assessed using Fisher’s exact test. *ns = non-significant.*

**1.14 Supplementary Figure 14**


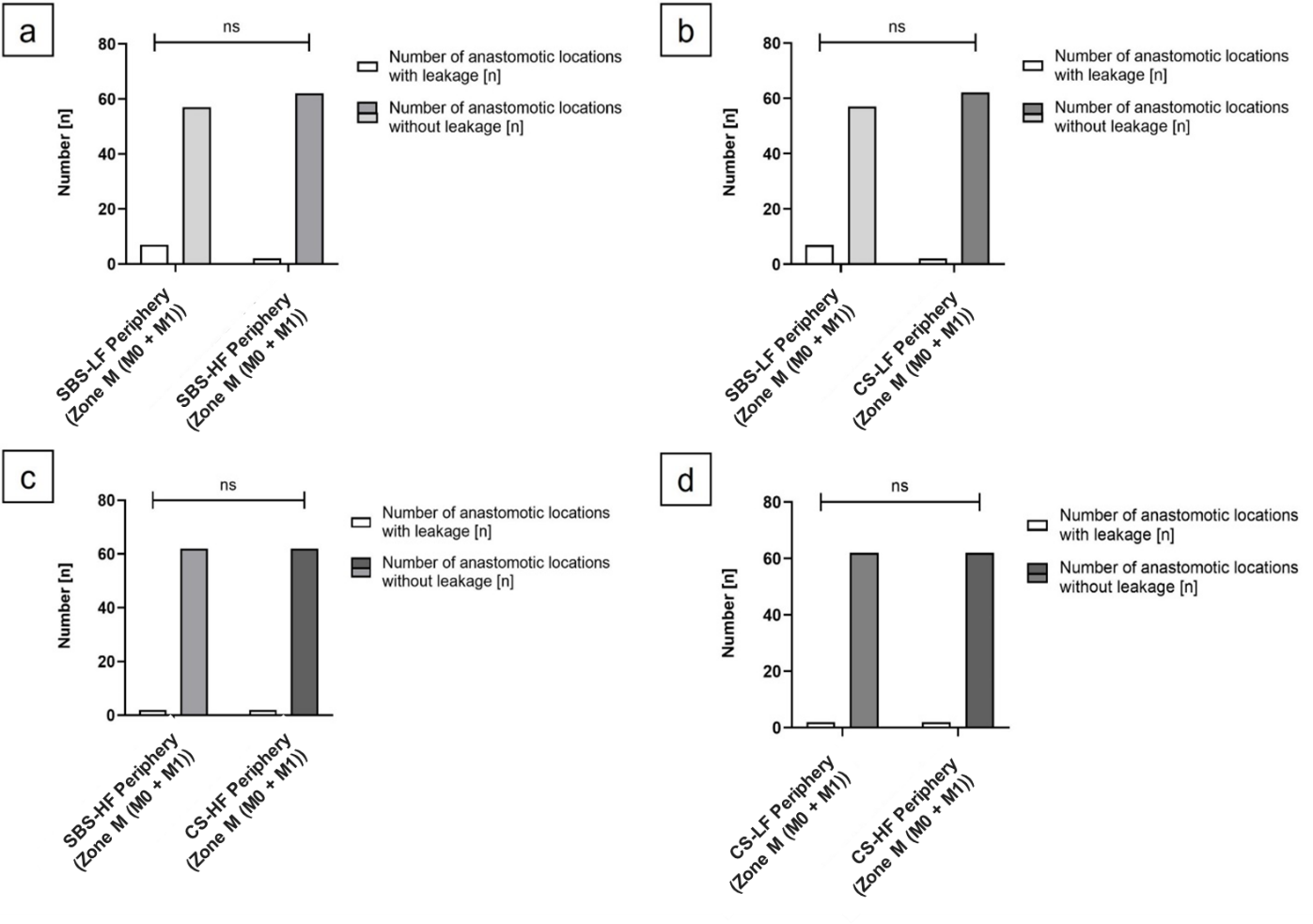


**Supplemetary Fig. 14 Differences in anastomotic leakage rates of areas within the peripheral zone among compared experimental series.** There were no statistically significant differences in the incidence of leakage at the peripheral zone between (a) SBS-LF and SBS-HF anastomoses ($p$ $= 0.1640$), (b) SBS-LF and CS-LF anastomoses ($p$ $= 0.1640$), (c) SBS-HF and CS-HF anastomoses ($p$ $> 0.9999$), and (d) CS-LF and CS-HF anastomoses ($p$ $> 0.9999$). Significance was assessed using Fisher’s exact test. *ns = non-significant*

**1.15 Supplementary Figure 15**


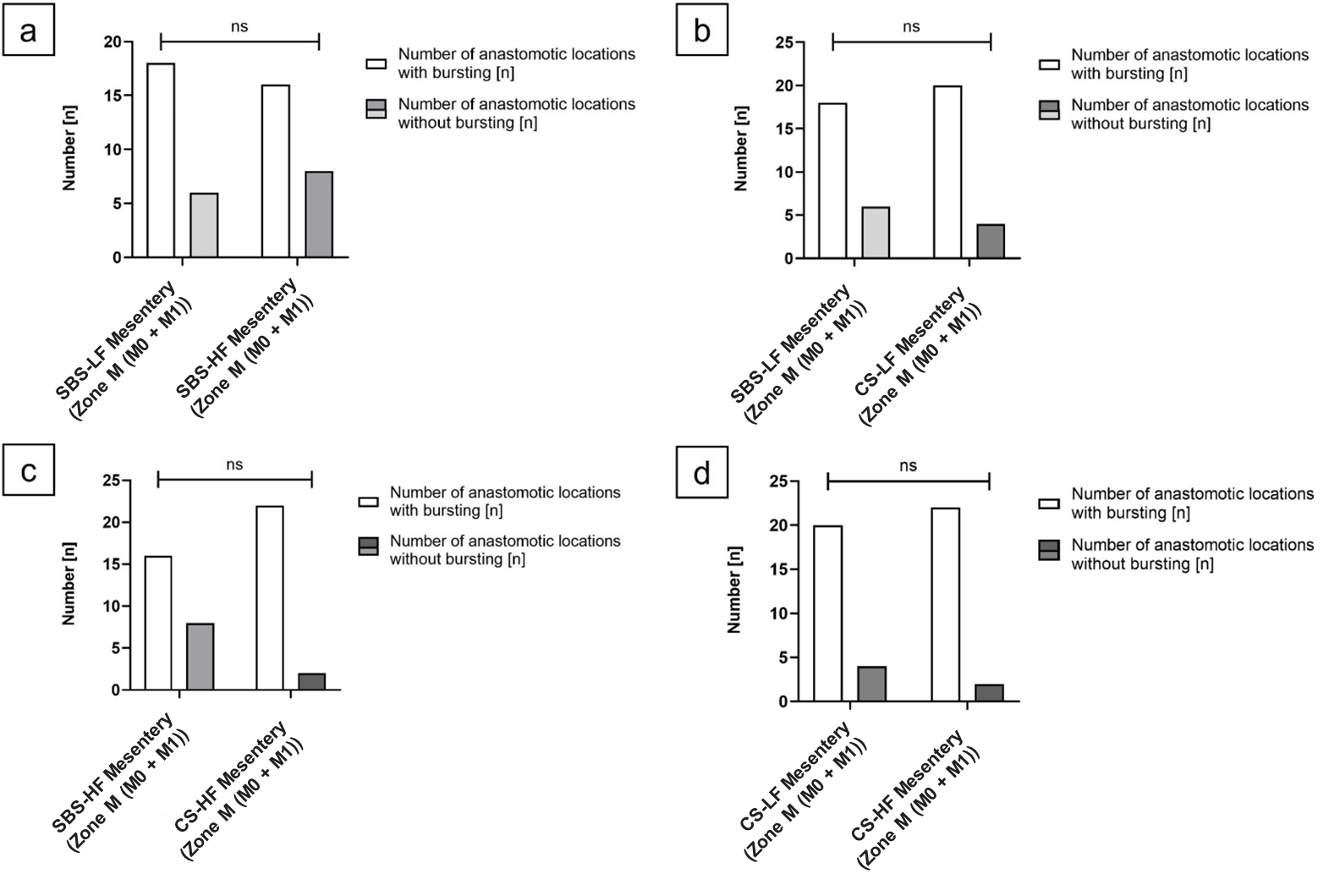


**Supplementary Fig. 15 Differences in anastomotic bursting rates of areas within the mesenteric zone among compared experimental series.** There were no statistically significant differences in the incidence of leakage at the mesenteric zone between (a) SBS-LF and SBS-HF anastomoses ($p$ $= 0.7516),$ (b) SBS-LF and CS-LF anastomoses ($p$ $= 0.6662$), (c) SBS-HF and CS-HF anastomoses ($p$ $= 0.0723$), and (d) CS-LF and CS-HF anastomoses ($p$ $= 0.6662$). Significance was assessed using Fisher’s exact test. *ns = non-significant*

**1.16 Supplementary Figure 16**


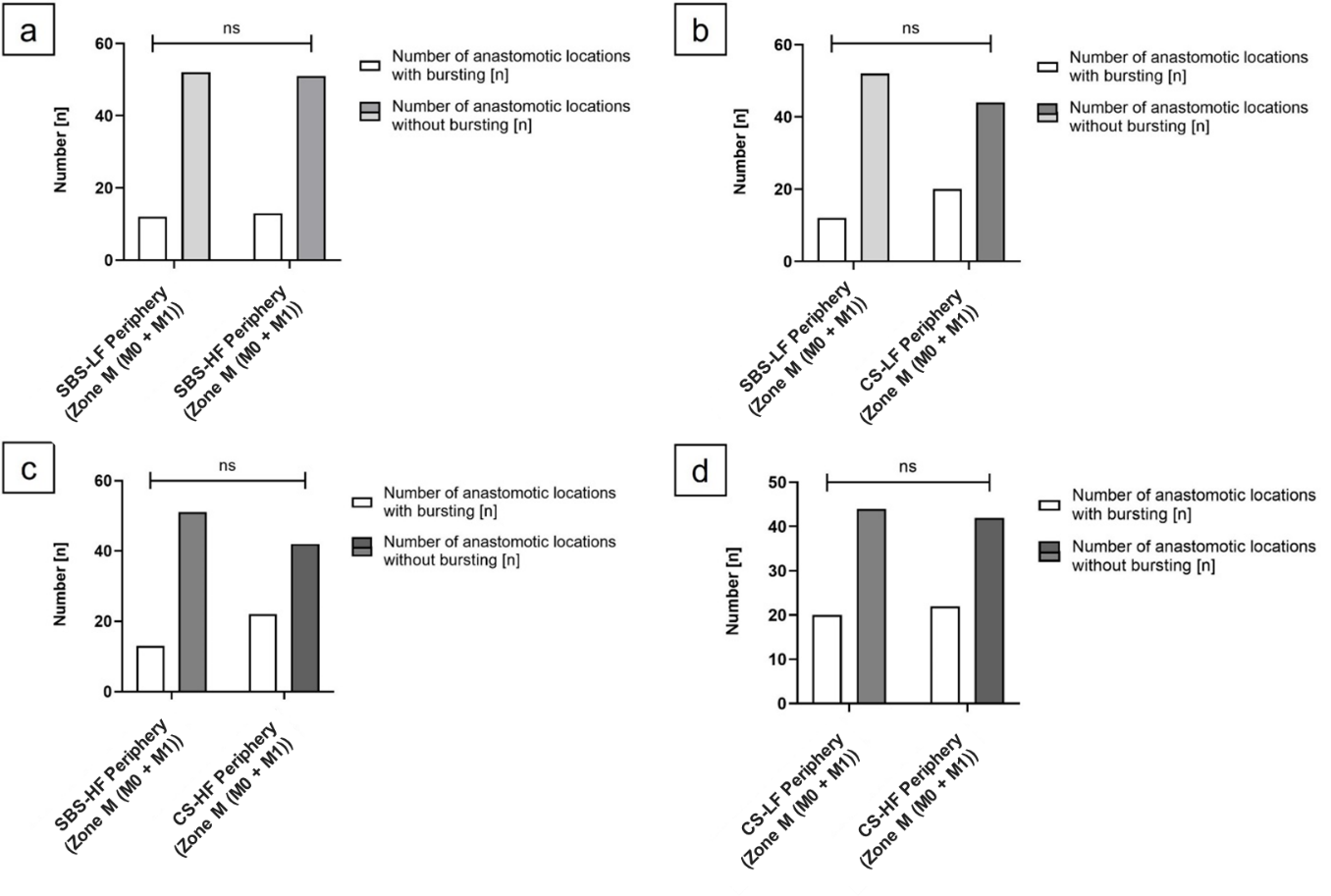


**Supplementary Fig. 16 Differences in anastomotic bursting rates of areas within the peripheral zone among compared experimental series.** There were no statistically significant differences in the incidence of leakage at the peripheral zone between (a) SBS-LF and SBS-HF anastomoses ($p > 0.9999$), (b) SBSLF and CS-LF anastomoses ($p$ $= 0.1524$), (c) SBS-HF and CS-HF anastomoses ($p$ $= 0.4866$), and (d) CS-LF and CS-HF anastomoses ($p$ $= 0.8508$). Significance was assessed using Fisher’s exact test. *ns = non-significant*

**2. Supplementary Tables**

**2.1 Supplementary Table 1**

| Supplementary Table 1. Innovative *ex-vivo* model. Chemicals, reagents, surgical and consumable materials, laboratory equipment, hardware and software. | | |
| --- | --- | --- |
| Type of material | **Name** | **Brand and supplier (postal code, city, country)** |
| Reagents | Distilled water | SAV Liquid Production GmbH  (83126 Flintsbach am Inn, Germany) |
|  | Formaldehyde solution  (PN: 252549) | Sigma-Aldrich Chemie GmbH  (82024 Taufkirchen, Germany) |
|  | Methyl green  (C. l. 42590) | Carl Roth GmbH & Co. KG  (76185 Karlsruhe, Germany) |
|  | Phosphate buffered solution tablets | EMD Millipore Corporation; Sigma-Aldrich Chemie GmbH (82024 Taufkirchen, Germany) |
| Parent solution | A 3.7% formaldehyde solution (1:9 ratio) was prepared by diluting 100 ml of ACS reagent (37 wt. % ) in water with 10-15% methanol as a stabilizer, in 900 ml of water. | See brand and supplier in [reagents](#Reagents) |
|  | A methyl green solution was prepared by dissolving 1 g of methyl green, one phosphate-buffered solution tablet, in 1 liter of distilled water. |  |
|  | A phosphate-buffered solution was made using 1 phosphate-buffered solution tablet and 1 liter of distilled water. |  |
| Surgical instruments and suture material | Anatomical forceps  (14.5 cm, ClinaStar) | Entrhal Medical GmbH  (47638 Straelen, Germany) |
|  | Disposable Scalpel  (Category number 11, 100 mm length) | FEATHER; SOCOREX ISBA SA  (1024 Ecublens/Lausanne, Switzerland) |
|  | Iris scissors  (11 cm, ClinaStar) | Entrhal Medical GmbH  (47638 Straelen, Germany) |
|  | Needle holder  (PN : 56127, Hegar-Mayo, MF: BM065R, 150 mm, wire 5/0 from Aesculap) | B. Braun SE  (34212 Melsungen, Germany) |
|  | Needle holder (Mayo-Hegar, 12 cm, ClinaStar) | Entrhal Medical GmbH  (47638 Straelen, Germany) |
|  | PDS II  (violet monofilament, with needle SH-1 PLUS (22 mm 1/2c and VISI-BLACK, USP 4-0, 70 cm thread length)) | Ethicon Deutschland Johnson & Johnson Medical GmbH  (22851 Norderstedt, Germany) |
| Other consumable materials | Cable ties (150 mm 2.50 mm Black UV-proof) | TRU COMPONENTS; Conrad Electronic SE  (1923 Berlin, Germany) |
|  | Connecting tubes:   - Tubing system with pressure converter (cut to size): Reduced roller pump tubing with connector AR-6411, Arthrex, no information on ID and WD, Self-measurement: ID = 4.8mm, WD = 1mm   Pump tubing: Tubing R-3603, TYGON, ID = 3.2mm, WD = 1.6mm (now replaced by model E-3603) | Arthrex GmbH  (81249 München, Germany)  TYGON (Saint-Gobain Performance Plastics)  (50769 Köln, Germany) |
|  | Custom-made Framework (Aluminum strut profiles of type 5 with 20 x 20 mm cross-section):   - Profile 5 20x20, natural, length: 58mm; Article number: 0.0.370.03 - Profile 5 20x20, natural, length: 180mm; Article number: 0.0.370.03 - Profile 5 20x20, natural, length: 210mm; Article number: 0.0.370.03 - Profile 5 20x20, natural, length: 400mm; Article number: 0.0.370.03 - Profile 5 20x20, natural, length: 450mm; Article number: 0.0.370.03 - Profile 5 20x20, natural, length: 368mm; Article number: 0.0.370.03 - Cover cap 5 20x20, black; Article number: 0.0.370.09 - Clamping lever Pi 50 M5x20, white; Article number: 0.0.678.50 - T-slot nut 5 St M5, galvanized; Article number: 0.0.370.01 - Automatic connection set 5, galvanized; Article number: 0.0.391.60 - Angle set 5 20x20; Article number: 0.0.425.02 - Joint 5 20x20 with clamping lever; Article number: 0.0.464.43 - Angle 5 20, white aluminum; Article number: 0.0.677.77 - Mounting set 5 2-4mm with countersunk M5; Article number: 0.0.680.92 - Half-round screw M5x16, galvanized; Article number: 8.0.000.07 - Half-round screw M5x12, galvanized; Article number: 8.0.005.45 - Angle 40x40x20 galvanized zinc; Article number: 0.0.474.60 - Clamping lever Pi 50 M5x16; Article number: 0.0.684.60 - Profile bar 5 St galvanized; Article number: 0.0.370.56   Angle set 5 20x20; Article number: 0.0.425.02 | Item Industrietechnik GmbH  (42651 Solingen, Germany) |
|  | Custom-made scaffolding walls (gray twin-wall sheet, ITEM (custom cut) |  |
|  | Cutting mat  (FINFÖRDELA – cutting mat, flexible, dark gray/dark turquoise, 28 x 36 cm) | IKEA Deutschland GmbH & Co. KG  (Germany) |
|  | Discofix C Three-Way Stopcock | B. Braun SE  (34212 Melsungen, Germany) |
|  | - Disposable pipettes (Transfer Pipets) | Avantor, Inc; VWR International GmbH  (64295 Darmstadt, Germany) |
|  | Duran Laboratory Bottle with Closure   - (1000 ml capacity, Borosilicate glass 3.3, polypropylene cap) | DURAN Schott; Avantor, Inc; VWR International GmbH  (64295 Darmstadt, Germany) |
|  | Flasks:   - Large: Duran Erlenmeyer flask, wide neck (2000 ml) - Small: Duran beaker (600 ml, borosilicate glass 3.3.) | DURAN Schott; Avantor, Inc; VWR International GmbH  (64295 Darmstadt, Germany) |
|  | Inject Luer-Solo Syringe (5 ml, 10 ml, 20 ml, 60 ml) | B. Braun SE  (34212 Melsungen, Germany) |
|  | Laboratory Lifting Platform | JUCHHEIM Laborgeräte GmbH  (79224 Umkirch, Germany) |
|  | Luer-Lock adapter:   - Adapter from male Luer-Lock to metric fitting PTFE-, PE for less elastic hoses / PC; ID 1.6 mm; rct-online; Thomafluid - Adapter from female Luer-Lock to metric fitting for less elastic hoses / PC; ID 1.6 mm; rct-online; Thomafluid - Adapter from male Luer-Lock to metric fitting PTFE-, PE for less elastic hoses / PC; ID 3.2 mm; rct-online; Thomafluid - Adapter from female Luer-Lock to metric fitting for less elastic hoses / PC; ID 3.2 mm; rct-online; Thomafluid - Adapter from male Luer-Lock to metric fitting PTFE-, PE for less elastic hoses / PC; ID 4.8 mm; rct-online; Thomafluid - Adapter from female Luer-Lock to metric fitting for less elastic hoses / PC; ID 4.8 mm; rct-online; Thomafluid | RCT Reichelt Chemietechnik GmbH + Co.  (69126 Heidelberg, Germany) |
|  | MoliNea E Water Absorbing Sheets | Paul Hartmann AG  (89522 Heidenheim, Germany) |
|  | Mounting for temperature probe, and different holder (made with 3D printer, Hose Center T1 v5, Hose Center T2 v4, Hose Holder I v2, Temperature Rod Holder Block v3, printed material from resin) |  |
|  | Materials for the perfusion bioreactor | The materials were adopted from Micheler et al., 2017 ^[^[1-5]^]^ |
|  | Ruler |  |
|  | Screw with a weight of 16,8 g |  |
|  | Specimen container  (Wide-mouth container, clear PVC, 500 ml) | Kautex Textron GmbH & Co KG  (80807 Munich, Germany) |
|  | LED ring light 60 | Omegon.de (86899 Landsberg am Lech, Germany) |
|  | StreamCam webcam | Logitech Europe S.A.  (1015 Lausanne, Switzerland) |
|  | USB-C hub 4 port | SITECOM Europe B.V.  (81274 CJ Huizen, The Netherlands) |
|  | NI-myRIO | National Instruments  (11500 Austin, USA) |
| Laboratory equipment | Digital heating plate (HP-20D Set) | Witeg Labortechnik GmbH  (97877 Wertheim, Germany) |
|  | GTH 175 Pt Digital Thermometer | Freisinger Electronic GmbH  (82031 Grünwald, Germany) |
|  | Magnetic stirrer with ceramic heating plate (180 x 180 mm, Professional Series | Avantor, Inc; VWR International GmbH (64295 Darmstadt, Germany) |
|  | Peristaltic pump (Ecoline VC-380/1, Ismatec) | Ismatec; VWR International GmbH  (64295 Darmstadt, Germany) |
|  | Pressure sensor (AMS 5812-0150-D) | Amsys GmbH & Co. KG (55124 Mainz, Germany) |
|  | Temperature sensor | Conrad Electronic SE (1923 Berlin, Germany) |
| Software Tools | LabView: LabVIEW 2021 SP1 f2 (16. February 2022) | National Instruments (11500 Austin, USA) |
|  | Office-Programs: Word, Excel, PowerPoint (Version 8.0.2) | Microsoft Corporation (San Diego, USA) |
|  | Endnote X9.3.1 (Build 13578)  Endnote 20.1 (Build 15341)  Endnote 21.2 (Build 17387) | Clarivate Analytics  (19130 Philadelphia, USA) |
|  | GraphPad Prism Software Version 8.0.2 | GraphPad Software, Inc.  (92108 San Diego, USA) |
|  | PhotoScape X 3.0.3 | MOOII TECH  (94111 San Francisco, USA) |
| *ACS = American Chemical Society; C. l. = Color index; ID = Inner diameter; MF = Manufacturer number; M5 = Metric thread size designation (5*$\boldsymbol{mm}$ *diameter); PC = Polycarbonate; PE = Polyethylene; PDS = Polydioxanone; PN = Product number; PTFE = Polytetrafluoroethylene; USP = United states pharmacopeia; WD = Wall thickness; wt. = Weight.* | | |

**2.2 Supplementary Table 2**

| **Supplementary Table 2.** **Innovative *ex-vivo* model.** Technologies and main components. | | |
| --- | --- | --- |
| **Technology or Main Component** | **Description** | **Figures** |
| Information Technology system | - HMI for user input (e.g. test parameters) - HMI inputs are transmitted to the NImyRIO controller - Data input and display of results via computer - Controller receives feedback signals from pressure sensor and records them (one measurement every 100 msec) - Temperature sensor is linked to controller for continuous monitoring and temperature regulation (maintain physiological body temperature in the sample chamber | **Figure 1 and Figure 2** |
| Fluid Power Technology | - Open fluid circulation system: peristaltic pump facilitates Methyl green solution from a reservoir into the anastomosis - Solution passes through the mechanical drive unit, sensor unit, test unit and anastomotic unit - Subsequent to the peristaltic pump, a pressure sensor with an integrated sensor chamber or adapter is installed (for measurement of generated hydrostatic pressure) - A temperature sensor is integrated to monitor the temperature of the medium and sample chamber - Program measurement and control technology of modified perfusion bioreactor with LabVIEW software - Transmission of program to controller for execution | **Figure 1 and Figure 2** |
| Anastomotic Unit | - stainless-steel screw (16.8 g) into the distal end of the anastomosis and fixation with two zip ties (prevention of leakage during testing and maintaining vertical position in the test chamber) - proximal end of anastomosis securely fasten with two zip ties to connection unit - The anastomotic unit is positioned in the PBS solution filled testing chamber (35 – 39 °C) - The three-way-valve is connected to the mechanical drive unit | **Figure 1;**  **Supplementary Figure 1;**  **Supplementary Figure 2** |
| Connection Unit | - Female Luer-Lock adapter connecting the end of a custom-cut tub to a three-way valve - Connection unit is flushed with methyl green-colored PBS solution prior testing (eliminate trapped air and ensure precise testing conditions) | **Supplementary Figure 2** |
| Test Unit | - Central component of the model - Consists of a sample chamber (custom-made transparent plastic square-shaped container with a single top opening) - The chamber is filled with 35 – 39 °C PBS solution (simulation of an intraabdominal physiological environment at body temperature) - A lifting platform aids in precise positioning during testing - The sample chamber is enclosed by a custom-made aluminum square-shaped frame featuring plastic walls with camera cutouts on each side - A manually operated lifting gate on the front facilitates easy access. An elevated frame above the chamber secures a ring light directly over the sample chamber - Electric wires are fastened along the frame to avoid contact with liquid materials - The fluid-transporting tube is connected to the anastomotic unit, and four cameras capture the experiment from different angles (25 frames per second with at least 2 frames per measurement) | **Figure 1.a, Figure 1.c,**  **Figure 1.d;**  **Figure 2;**  **Supplementary Figure 1;**  **Supplementary Figure 2.e** |
| Sensor Unit | - Consists of a pressure sensor temperature sensor within a watertight box with an opening for pressure equalization - A temperature probe within the sample chamber ensures continuous temperature monitoring - The pressure sensor is linked to the fluid-transporting tube, accurately measuring intraluminal pressure | **Figure 1.a, Figure 1.c, Figure 1.d; Figure 2** |
| Mechanical Drive Unit | - Consist of an external peristaltic pump which facilitates fluid (methyl green-colored PBS solution at 35 – 39 °C) flow to the anastomosis via the fluid-transporting tube, after passing the pressure sensor and connection unit, forming an open circulatory system - Temperature maintenance is ensured by a heater | **Figure 1.c, Figure 1.d; Figure 2;**  **Supplementary Figure 1; Supplementary Figure 2** |
| Control Unit | - The control unit has been customized into an automated, software-controlled system with a HMI to - Simultaneous recordings of intraluminal pressure within the tube and the anastomosis, as well as the surrounding temperature of the anastomosis within the sample chamber, while capturing camera footage - The operations of the adapted bioreactor are overseen and regulated by the real-time capable NI-myRIO controller (programmed using LabVIEW development environment) | **Figure 3** |
| *^a^ adapted and modified from Micheler et al. [3-5]*  *g = gram; HMI = Human Machine Interface; LabVIEW = Laboratory Virtual Instrumentation Engineering Workbench (LabVIEW 2021 SP1 f2, National Instruments, Austin, USA) software; msec = millisecond; PBS = Phosphate Buffered Saline Solution; °C = degree Celsius.* | | |

**2.3 Supplementary Table 3**

| Supplementary Table 3. Innovative *ex-vivo* model. Data Analysis. | | |
| --- | --- | --- |
| Parameter | **Content and Definition** | **Program** |
| Measured parameters | - Date, time stamp, runtime of the experiment (msec) - Temperature (°C) - Pressure (mmHg), pressure difference (mmHg), mean pressure (mmHg), pressure temperature (°C) - Image files (automatically saved and sorted based on the order of acquisition, with filenames generated automatically according to timestamps) | Microsoft Excel |
| Runtime | - Standardized to zero - The first pressure difference value closest to the acquired time stamp was identified - The value was required to be ≥ 0 and not to be followed by negative values - When leakage occurred at the start of the test, negative pressure difference values were accepted | Microsoft Excel |
| Visualization of pressure difference data | - The pressure difference was represented by the y-axis and the runtime zeroed (msec) was represented by the x-axis - For the purpose of transforming and reviewing the images in a video format, a Video converter was programmed in LabView, facilitating efficient image processing and visualization | GraphPad Prism Software |
| *msec = milliseconds; LabVIEW = Laboratory Virtual Instrumentation Engineering Workbench (LabVIEW 2021 SP1 f2, National Instruments, Austin, USA) software; mmHg = millimeter of mercury; °C = degree Celsius; ≥ = greater or equal.* | | |

**2.4 Supplementary Table 4**

| Supplementary Table 4. Innovative *ex-vivo* model. Quantitative analysis of anastomotic performance and time intervals. Experimental parameters, definitions and data acquisition procedures. | | |
| --- | --- | --- |
| Parameter | **Definition** | **Procedure for Data Acquisition** |
| Start pressure (Figure 4) | Pressure difference measured at the start of an experiment. | - Analysis of acquired images and data for identification of time stamp corresponding to the start of the experiment. - Pressure difference value closest to acquired time stamp, ≥ 0 and not followed by negative values, was identified in the data sheet. - Exception: if leakage occurred at the start of the test, negative pressure differences were accepted as start pressure, because it was assumed that the outflow from the anastomotic lumen might outweigh the influx, which leads to a temporary negative intraluminal pressure. |
| LP (Figure 4) | Pressure difference measured at first visible AL of an experiment | - All acquired images from all four cameras were manually screened for the first visible leakage. - If the first visible leakage was detected on images from one camera view, the corresponding pressure difference was extracted from the data sheet (time stamp). - The time stamps closest to the previously mentioned was utilized to select images from the other camera views. |
| BP (Figure 4) | Peak pressure difference recorded during an experiment. | - The dataset’s highest value of pressure difference was obtained by utilizing the “MAX” function in Microsoft Excel. - Manual screening of data and images was performed for verification of an actual bursting event. - The time stamp associated with the highest pressure difference was utilized to select images from one camera view. - The time stamps closest to the previously mentioned was utilized to select images from the other camera views. |
| Time interval analysis (Figure 4) | Analysis of the temporal dynamics associated with LP and BP (numerical; msec) | - Start to LP Time: time interval between the start of the experiment and LP - Start to BP Time: time interval between the start of the experiment and BP - LP to BP Time: time interval between LP and BP |
| *AL = Anastomotic leakage; BP = Bursting pressure; LP = Leakage pressure; msec = milliseconds; ≥ = greater or equal.* | | |

**2.5 Supplementary Table 5**

| Supplementary Table 5. Innovative *ex-vivo* model. Interrelated analyses derived from experimental outcome. Experimental parameters, definitions, data acquisition procedures and significance. | | |
| --- | --- | --- |
| Parameter | **Definition** | **Procedure for Data Acquisition and Significance** |
| Proportion of BP at LP | Quantification of the ratio of BP to LP, measured in mmHg (provided insights into the pressure dynamics between LP and BP) | - The proportion of LP and BP were calculated with absolute numerical values (mmHg) of measured pressure differences using Microsoft Excel to assess for the pressure increase from LP to BP. - This parameter offers insights into pressure the pressure relationship between LP and BP, allowing for direct comparison of pressure differences between experimental groups/ series. |
| Percentage of BP at LP | Quantification of the proportion of recorded pressure difference at BP in relation to recorded pressure difference at LP in %. | - The % of pressure difference at BP at pressure difference at LP was calculated as a relative measure using Microsoft Excel, to assess for the relative proportion of pressure difference at BP reached at the pressure difference of LP. - This parameter offers insights into the relative magnitude of pressure values associated with LP and BP and allows for comparison the proportion of pressure difference at BP achieved relative to pressure difference at LP (by normalizing the BP based on the individual LP). - Standardized assessment of BP in the context of LP to study pressure dynamics during LP and BP |
| Relative Difference of Pressure between LP and BP | Quantification of the pressure gab between LP and BP in % | - Ther relative pressure difference between LP and BP (%) was calculated using Microsoft Excel. - This parameter offers insights into %-based pressure fluctuations or variability between LP and BP. |
| Time of LP Relative to BP time | Quantification of temporal relationship between LP and BP in msec. | - The proportion of start to LP time relative to start to BP time (msec) was calculated using Microsoft Excel. - This parameter offers insights into the sequence and temporal proximity of LP and BP throughout the experiment and allows for comparison of absolute time needed to reach BP after LP occurs between experimental groups/ series. |
| % of time of BP at time of LP | Quantification of proportion of time spent at BP in relation to time spent at LP in %. | - The proportion of time spent at BP relative to time spent at LP (%) was calculated using Microsoft Excel. - This parameter offers insights into the sequence and temporal proximity of LP and BP and enables comparison between experimental groups/ series. |
| Relative Difference of Time between LP and BP | Quantification of proportion of time spent at BP concerning time elapsed between LP and BP in %. | - The proportion of time spent at BP to time elapsed between LP and BP (%) was calculated using Microsoft Excel. - This parameter provides a relative measure of the duration at BP during the occurrence of LP, compared to the overall time spent at LP |
| *BP = Bursting pressure; LP = Leakage pressure; mmHg = millimeters of mercury; % = Percent.* | | |

**2.6 Supplementary Table 6**

| Supplementary Table 6. Quantitative analysis of anastomotic performance: Evaluation of leakage pressure and bursting pressure. Summary of descriptive statistics for the SBS-LF experimental series. | | |
| --- | --- | --- |
| Anastomosis (name) | **Leakage pressure (LP) in mmHg** | **Bursting pressure (BP) in mmHg** |
| SBS-LF-n1 | -0.4 | 8.9 |
| SBS-LF-n2 | 32.3 | 100.1 |
| SBS-LF-n3 | 42.2 | 179.3 |
| SBS-LF-n4 | 8.9 | 41.5 |
| SBS-LF-n5 | 12.8 | 67.4 |
| SBS-LF-n6 | 14.1 | 128.2 |
| SBS-LF-n7 | 24.2 | 47.3 |
| SBS-LF-n8 | 1.7 | 145.2 |
| Summary of descriptive statistics | | |
| Mean | 16.98 | 89.74 |
| Standard error (*SEM*) | 5.26 | 20.64 |
| Median | 13.45 | 83.75 |
| Mode | N/A | N/A |
| Standard deviation (*SD*) | 14.88 | 58.37 |
| Sample variance | 221.35 | 3,406.53 |
| Kurtosis | -0.61 | -1.13 |
| Skewness | 0.61 | 0.21 |
| Range | 42.6 | 170.4 |
| Minimum | -0.4 | 8.9 |
| Maximum | 42.2 | 179.3 |
| Sum | 135.8 | 717.9 |
| Count | 8 | 8 |
| Confidence level (*CI*) (95 %) | 12.44 | 48.80 |
| Upper *CI* (95 %) | 29.41 | 138.53 |
| Lower *CI* (95 %) | 4.54 | 40.94 |
| *SBS-LF = Handsewn sufficient small intestinal end-to-end anastomoses using interrupted suture technique, tested in the low flow model; mmHg = Millimeters of mercury; N/A = Not available.* | | |

**2.7 Supplementary Table 7**

| Supplementary Table 7. Quantitative analysis of anastomotic performance: Evaluation of leakage pressure and bursting pressure. Summary of descriptive statistics for the SBS-HF experimental series. | | |
| --- | --- | --- |
| Anastomosis (name) | **Leakage pressure (LP) in mmHg** | **Bursting pressure (BP) in mmHg** |
| SBS-HF-n1 | 5.9 | 149.3 |
| SBS-HF-n2 | 66.8 | 166.7 |
| SBS-HF-n3 | 81.1 | 156.5 |
| SBS-HF-n4 | 43.3 | 201.9 |
| SBS-HF-n5 | 154.2 | 194.5 |
| SBS-HF-n6 | 103.7 | 145.2 |
| SBS-HF-n7 | 24.9 | 210.9 |
| SBS-HF-n8 | 19 | 86 |
| Summary of descriptive statistics | | |
| Mean | 62.36 | 163.88 |
| Standard error (*SEM*) | 17.60 | 14.20 |
| Median | 55.05 | 161.6 |
| Mode | N/A | N/A |
| Standard deviation (*SD*) | 49.78 | 40.15 |
| Sample variance | 2,478.41 | 1,611.89 |
| Kurtosis | 0.12 | 1.01 |
| Skewness | 0.82 | -0.87 |
| Range | 148.3 | 124.9 |
| Minimum | 5.9 | 86 |
| Maximum | 154.2 | 210.9 |
| Sum | 4,989 | 1,311 |
| Count | 8 | 8 |
| Confidence level (*CI*) (95 %) | 41.62 | 33.57 |
| Upper *CI* (95 %) | 103.98 | 197.44 |
| Lower *CI* (95 %) | 20.74 | 130.31 |
| *SBS-HF = Handsewn sufficient small intestinal end-to-end anastomoses using interrupted suture technique, tested in the high-flow model; mmHg = Millimeters of mercury; N/A = Not available.* | | |

**2.8 Supplementary Table 8**

| Supplementary Table 8. Quantitative analysis of anastomotic performance: Evaluation of leakage pressure and bursting pressure. Summary of descriptive statistics for the CS-LF experimental series. | | |
| --- | --- | --- |
| Anastomosis (name) | **Leakage pressure (LP) in mmHg** | **Bursting pressure (BP) in mmHg** |
| CS-LF-n1 | 17.2 | 34.7 |
| CS-LF-n2 | 73 | 122.3 |
| CS-LF-n3 | 30.9 | 66.3 |
| CS-LF-n4 | 43.8 | 76.7 |
| CS-LF-n5 | 47.9 | 74.6 |
| CS-LF-n6 | 13.5 | 98.4 |
| CS-LF-n7 | 7.6 | 72.2 |
| CS-LF-n8 | 45.2 | 87.7 |
| Summary of descriptive statistics | | |
| Mean | 34.89 | 79.11 |
| Standard error (*SEM*) | 7.73 | 8.99 |
| Median | 37.35 | 75.65 |
| Mode | N/A | N/A |
| Standard deviation (*SD*) | 21.84 | 25.42 |
| Sample variance | 476.92 | 645.93 |
| Kurtosis | -0.35 | 1.30 |
| Skewness | 0.43 | -0.02 |
| Range | 65.4 | 87.6 |
| Minimum | 7.6 | 34.7 |
| Maximum | 73 | 122.3 |
| Sum | 279.1 | 632.9 |
| Count | 8 | 8 |
| Confidence level (*CI*) (95 %) | 18.26 | 21.25 |
| Upper *CI* (95 %) | 53.15 | 100.36 |
| Lower *CI* (95 %) | 16.63 | 57.87 |
| *CS-LF = Handsewn sufficient small intestinal end-to-end anastomoses using simple continuous technique, tested in the low-flow model; mmHg = Millimeters of mercury; N/A = Not available.* | | |

**2.9 Supplementary Table 9**

| Supplementary Table 9. Quantitative analysis of anastomotic performance: Evaluation of leakage pressure and bursting pressure. Summary of descriptive statistics for the CS-HF experimental series. | | |
| --- | --- | --- |
| Anastomosis (name) | **Leakage pressure (LP) in mmHg** | **Bursting pressure (BP) in mmHg** |
| CS-HF-n1 | 22.5 | 131.2 |
| CS-HF-n2 | 70 | 150.5 |
| CS-HF-n3 | 20 | 138.4 |
| CS-HF-n4 | 98.7 | 184.7 |
| CS-HF-n5 | 28 | 131.8 |
| CS-HF-n6 | 21.7 | 190 |
| CS-HF-n7 | 24.2 | 214.7 |
| CS-HF-n8 | 7.6 | 211.7 |
| Summary of descriptive statistics | | |
| Mean | 36.59 | 169.13 |
| Standard error (*SEM*) | 10.97 | 12.46 |
| Median | 23.35 | 167.6 |
| Mode | N/A | N/A |
| Standard deviation (*SD*) | 31.02 | 35.24 |
| Sample variance | 962.44 | 1,241.49 |
| Kurtosis | 1.40 | -2.04 |
| Skewness | 1.53 | 0.19 |
| Range | 91.1 | 83.5 |
| Minimum | 7.6 | 131.2 |
| Maximum | 98.7 | 214.7 |
| Sum | 292.7 | 1,353 |
| Count | 8 | 8 |
| Confidence level (*CI*) (95 %) | 25.94 | 29.46 |
| Upper *CI* (95 %) | 62.52 | 198.58 |
| Lower *CI* (95 %) | 10.65 | 139.67 |
| *CS-HF = Handsewn sufficient small intestinal end-to-end anastomoses using simple continuous technique, tested in the high-flow model; mmHg = Millimeters of mercury; N/A = Not available.* | | |

**2.10 Supplementary Table 10**

| Supplementary Table 10. Quantitative analysis of anastomotic performance in the SBS-LF experimental series: Time interval analysis. Interrelated analyses derived from experimental outcome. | | | | | | | | |
| --- | --- | --- | --- | --- | --- | --- | --- | --- |
| Anastomosis (name) | **Time**  **(start to leakage pressure),**  **in msec** | **Time (start to bursting pressure), in msec** | **Time (LP to BP), in msec** | **Proportion of BP at LP, in mmHg** | **Percentage of BP at LP,**  **in %** | **Relative difference of pressure between LP and BP, in %** | **Time of leakage occurrence relative to bursting time,**  **in %** | **Relative difference of time between leakage and bursting time,**  **in %** |
| SBS-LF-n1 | 6,800 | 25,300 | 18,500 | 9.30 | -4.49 | 104.49 | 26.88 | 73.12 |
| SBS-LF-n2 | 43,169 | 279,869 | 236,700 | 67.80 | 32.27 | 67.73 | 15.43 | 84.58 |
| SBS-LF-n3 | 14,249 | 56,700 | 42,451 | 137.10 | 23.54 | 76.46 | 25.13 | 74.87 |
| SBS-LF-n4 | 26,729 | 62,002 | 35,273 | 32.60 | 21.45 | 78.55 | 43.11 | 56.89 |
| SBS-LF-n5 | 38,302 | 124,805 | 86,503 | 54.60 | 18.99 | 81.01 | 30.69 | 69.31 |
| SBS-LF-n6 | 37,513 | 124,099 | 86,586 | 114.10 | 11.00 | 89.00 | 30.23 | 69.77 |
| SBS-LF-n7 | 21,304 | 61,802 | 40,498 | 23.10 | 51.16 | 48.84 | 34.47 | 65.53 |
| SBS-LF-n8 | 14,103 | 2,983,020 | 284,199 | 143.50 | 1.17 | 98.83 | 4.73 | 95.27 |
| Summary of descriptive statistics | | | | | | | | |
| Mean | 25,271.13 | 129,109.88 | 103,838.75 | 72.76 | 19.39 | 80.62 | 26.33 | 73.67 |
| Standard error (*SEM*) | 4,715.59 | 36,948.67 | 35,492.60 | 18.57 | 6.23 | 6.23 | 4.16 | 4.16 |
| Median | 24,016.5 | 93,050.5 | 64,477 | 61.2 | 20.22 | 79.78 | 28.55 | 71.45 |
| Mode | N/A | N/A | N/A | N/A | N/A | N/A | N/A | N/A |
| Standard deviation (*SD*) | 13,337.71 | 104,506.61 | 100,388.24 | 52.53 | 17.61 | 17.61 | 11.77 | 11.77 |
| Sample variance | 177,894,475.8 | 10,921,632,403 | 10,077,798,275 | 2,759.04 | 309.96 | 309.96 | 138.48 | 138.48 |
| Kurtosis | -1.64 | -0.53 | 0.08 | -1.74 | 0.41 | 0.41 | 0.75 | 0.75 |
| Skewness | 0.06 | 1.03 | 1.28 | 0.32 | 0,49 | -0.49 | -0.70 | 0.70 |
| Range | 36,369 | 273,002 | 265,699 | 134.2 | 55.66 | 55.66 | 38.38 | 38.38 |
| Minimum | 6,800 | 25,300 | 18,500 | 9.3 | -4.49 | 48.84 | 4.73 | 56.89 |
| Maximum | 43,169 | 298,302 | 284,199 | 143.5 | 51.16 | 104.49 | 43.11 | 95.27 |
| Sum | 202,169 | 1,032,879 | 830,710 | 582.1 | 155.08 | 644.92 | 210.66 | 589.34 |
| Count | 8 | 8 | 8 | 8 | 8 | 8 | 8 | 8 |
| Confidence level (*CI*) (95 %) | 11,150.60 | 87,369.72 | 83,926.67 | 43.91 | 14.72 | 14.72 | 9.84 | 9.84 |
| Upper *CI* (95 %) | 36,421.73 | 216,479.59 | 187,765.42 | 116.68 | 34.10 | 95.33 | 36.17 | 83.51 |
| Lower *CI* (95 %) | 14,120.52 | 41,740.16 | 19,912.08 | 28.85 | 4.67 | 65.90 | 16.49 | 63.83 |
| *SBS-HF = Handsewn sufficient small intestinal end-to-end anastomoses using interrupted suture technique, tested in the high-flow model; mmHg = Millimeters of mercury; msec = Milliseconds; % = Percent; N/A = Not available.* | | | | | | | | |

**2.11 Supplementary Table 11**

| Supplementary Table 11. Quantitative analysis of anastomotic performance in the SBS-LF experimental series: Time interval analysis. Interrelated analyses derived from experimental outcome. | | | | | | | | |
| --- | --- | --- | --- | --- | --- | --- | --- | --- |
| Anastomosis (name) | **Time**  **(start to leakage pressure),**  **In msec** | **Time (start to bursting pressure), in msec** | **Time (LP to BP), in msec** | **Proportion of BP at LP, in mmHg** | **Percentage of BP at LP,**  **in %** | **Relative difference of pressure between LP and BP, in %** | **Time of leakage occurrence relative to bursting time,**  **in %** | **Relative difference of time between leakage and bursting time,**  **in %** |
| SBS-HF-n1 | 2,004 | 19,900 | 17,896 | 143.40 | 3.95 | 96.05 | 10.07 | 89.93 |
| SBS-HF-n2 | 7,712 | 27,899 | 20,187 | 99.90 | 40.07 | 59.93 | 27.64 | 72.36 |
| SBS-HF-n3 | 8,401 | 20,200 | 11,799 | 75.40 | 51.82 | 48.18 | 41.59 | 58.41 |
| SBS-HF-n4 | 2,499 | 46,799 | 44,300 | 158.60 | 21.45 | 78.55 | 5.34 | 94.66 |
| SBS-HF-n5 | 10,585 | 20,184 | 9,599 | 40.30 | 79.28 | 20.72 | 52.44 | 47.56 |
| SBS-HF-n6 | 5,704 | 32,098 | 26,394 | 41.50 | 71.42 | 28.58 | 17.77 | 82.23 |
| SBS-HF-n7 | 1,688 | 24,088 | 22,400 | 186.00 | 11.81 | 88.19 | 7.01 | 92.99 |
| SBS-HF-n8 | 1,870 | 9,560 | 7,690 | 67.00 | 22.09 | 77.91 | 19.56 | 80.44 |
| Summary of descriptive statistics | | | | | | | | |
| Mean | 5,057.88 | 25,091 | 5,057.88 | 101.51 | 37.74 | 62.26 | 22.68 | 77.32 |
| Standard error (*SEM*) | 1,243.41 | 3,885.38 | 1,243.41 | 19.54 | 9.81 | 9.81 | 5.99 | 5.99 |
| Median | 4,101.5 | 22,144 | 4,101.5 | 87.65 | 31.08 | 68.92 | 18.67 | 81.33 |
| Mode | N/A | N/A | N/A | N/A | N/A | N/A | N/A | N/A |
| Standard deviation (*SD*) | 3,516.90 | 10,989.5090 | 3,516.90 | 55.26 | 27.74 | 27.75 | 16.93 | 16.93 |
| Sample variance | 12,368,578.7 | 120,769,308.3 | 12,368,578.7 | 3,053.68 | 769.72 | 769.72 | 286.73 | 286.73 |
| Kurtosis | -1.61 | 1.7901 | -1.61 | -1.46 | -1.32 | -1.32 | -0.36 | -0.36 |
| Skewness | 0.48 | 0.93 | 0.49 | 0.40 | 0.43 | -0.43 | 0.87 | -0.87 |
| Range | 8,897 | 37,239 | 8,897 | 145.7 | 75.33 | 75.33 | 47.10 | 47.10 |
| Minimum | 1,688 | 9,560 | 1,688 | 40.3 | 3.95 | 20.72 | 5.34 | 47.56 |
| Maximum | 10,585 | 46,799 | 10,585 | 186 | 79.28 | 96.05 | 52.44 | 94.66 |
| Sum | 40,463 | 200,728 | 40,463 | 812.1 | 301.89 | 498.11 | 181.42 | 618.58 |
| Count | 8 | 8 | 8 | 8 | 8 | 8 | 8 | 8 |
| Confidence level (*CI*) (95 %) | 2,940.20 | 9,187.46 | 2,940.20 | 46.20 | 23.20 | 23.20 | 14.16 | 14.16 |
| Upper *CI* (95 %) | 7,998.08 | 34,278.46 | 7,998.08 | 147.71 | 60.93 | 85.46 | 36.83 | 91.48 |
| Lower *CI* (95 %) | 2,117.67 | 15,903.54 | 2,117.67 | 55.31 | 14.54 | 39.07 | 8.52 | 63.17 |
| *SBS-HF = Handsewn sufficient small intestinal end-to-end anastomoses using interrupted suture technique, tested in the high-flow model; mmHg = Millimeters of mercury; msec = Milliseconds; % = Percent; N/A = Not available.* | | | | | | | | |

**2.12 Supplementary Table 12**

| Supplementary Table 12. Quantitative analysis of anastomotic performance in the CS-LF experimental series: Time interval analysis. Interrelated analyses derived from experimental outcome. | | | | | | | | |
| --- | --- | --- | --- | --- | --- | --- | --- | --- |
| Anastomosis (name) | **Time**  **(start to leakage pressure),**  **In msec** | **Time (start to bursting pressure), in msec** | **Time (LP to BP), in msec** | **Proportion of BP at LP, in mmHg** | **Percentage of BP at LP,**  **in %** | **Relative difference of pressure between LP and BP, in %** | **Time of leakage occurrence relative to bursting time,**  **in %** | **Relative difference of time between leakage and bursting time,**  **in %** |
| CS-LF-n1 | 14,941 | 57,941 | 43,000 | 17.5 | 49.57 | 50.43 | 25.79 | 74.21 |
| CS-LF-n2 | 56,502 | 212,706 | 156,204 | 49.3 | 59.69 | 40.31 | 26.56 | 73.44 |
| CS-LF-n3 | 35,300 | 75,002 | 39,702 | 35.4 | 46.61 | 53.39 | 47.07 | 52.94 |
| CS-LF-n4 | 34,700 | 131,702 | 97,002 | 32.9 | 57.11 | 42.89 | 26.35 | 73.65 |
| CS-LF-n5 | 69,300 | 130,510 | 61,210 | 26.7 | 64.21 | 35.79 | 53.10 | 46.90 |
| CS-LF-n6 | 25,893 | 88,680 | 62,787 | 84.9 | 13.72 | 86.28 | 29.20 | 70.80 |
| CS-LF-n7 | 5,508 | 129,200 | 123,692 | 64.6 | 10.53 | 89.47 | 4.26 | 95.74 |
| CS-LF-n8 | 26,405 | 56,297 | 29,892 | 42.5 | 51.54 | 48.46 | 46.90 | 53.10 |
| Summary of descriptive statistics | | | | | | | | |
| Mean | 33,568.63 | 110,254.75 | 76,686.13 | 44.23 | 44.12 | 55.88 | 32.40 | 67.60 |
| Standard error (*SEM*) | 7,381.89 | 18,459.34 | 15,851.06 | 7.71 | 7.27 | 7.27 | 5.62 | 5.62 |
| Median | 30,552.5 | 108,940 | 61,998.5 | 38.95 | 50.55 | 49.45 | 27.88 | 72.12 |
| Mode | N/A | N/A | N/A | N/A | N/A | N/A | N/A | N/A |
| Standard deviation (*SD*) | 20,879.14 | 52,210.88 | 44,833.58 | 21.80 | 20.56 | 20.56 | 15.90 | 15.90 |
| Sample variance | 435,938,335.4 | 2,725,976,322 | 2,010,049,469 | 475.12 | 422.61 | 422.61 | 252.89 | 252.89 |
| Kurtosis | -0.1 | 1.03 | -0.40 | 0.47 | -0.33 | -0.33 | -0.05 | -0.05 |
| Skewness | 0.59 | 0.9987 | 0.88 | 0.91 | -1.13 | 1.13 | -0.38 | 0.38 |
| Range | 63,792 | 156,409 | 126,312 | 67.4 | 53.68 | 53.68 | 48.84 | 48.84 |
| Minimum | 5,508 | 56,297 | 29,892 | 17.5 | 10.53 | 35.79 | 4.26 | 46.90 |
| Maximum | 69,300 | 212,706 | 156,204 | 84.9 | 64.21 | 89.47 | 53.10 | 95.74 |
| Sum | 268,549 | 882,038 | 613,489 | 353.8 | 352.96 | 447.04 | 259.23 | 540.77 |
| Count | 8 | 8 | 8 | 8 | 8 | 8 | 8 | 8 |
| Confidence level (*CI*) (95 %) | 17,455.40 | 43,649.39 | 37,481.81 | 18.22 | 17.19 | 17.19 | 13.30 | 13.30 |
| Upper *CI* (95 %) | 51,024.02 | 153,904.14 | 114,167.93 | 62.45 | 61.31 | 73.07 | 45.70 | 80.89 |
| Lower *CI* (95 %) | 16,113.23 | 66,605.36 | 39,204.32 | 26.00 | 26.93 | 38.69 | 19.11 | 54.30 |
| *CS-LF = Handsewn sufficient small intestinal end-to-end anastomoses using simple continuous suture technique, tested in the low-flow model; mmHg = Millimeters of mercury; msec = Milliseconds; % = Percent; N/A = Not available.* | | | | | | | | |

**2.13 Supplementary Table 13**

| Supplementary Table 13. Quantitative analysis of anastomotic performance in the CS-HF experimental series: Time interval analysis. Interrelated analyses derived from experimental outcomes. | | | | | | | | |
| --- | --- | --- | --- | --- | --- | --- | --- | --- |
| Anastomosis (name) | **Time**  **(start to leakage pressure),**  **In msec** | **Time (start to bursting pressure), in msec** | **Time (LP to BP), in msec** | **Proportion of BP at LP, in mmHg** | **Percentage of BP at LP,**  **in %** | **Relative difference of pressure between LP and BP, in %** | **Time of leakage occurrence relative to bursting time,**  **in %** | **Relative difference of time between leakage and bursting time,**  **in %** |
| CS-HF-n1 | 33,602 | 36,893 | 33,602 | 108.7 | 17.15 | 82.85 | 8.92 | 91.08 |
| CS-HF-n2 | 23,699 | 31,956 | 23,699 | 80.5 | 46.51 | 53.49 | 25.84 | 74.16 |
| CS-HF-n3 | 71,400 | 73,593 | 71,400 | 118.4 | 14.45 | 85.55 | 2.98 | 97.02 |
| CS-HF-n4 | 20,328 | 28,129 | 20,328 | 86 | 53.44 | 46.56 | 27.73 | 72.27 |
| CS-HF-n5 | 80,089 | 82,499 | 80,089 | 103.8 | 21.24 | 78.76 | 2.92 | 97.08 |
| CS-HF-n6 | 60,001 | 64,001 | 60,001 | 168.3 | 11.42 | 88.58 | 6.25 | 93.75 |
| CS-HF-n7 | 25,500 | 27,600 | 25,500 | 190.5 | 11.27 | 88.73 | 7.61 | 92.39 |
| CS-HF-n8 | 18,002 | 18,002 | 18,002 | 204.1 | 3.59 | 96.41 | 0 | 100 |
| Summary of descriptive statistics | | | | | | | | |
| Mean | 3,756.5 | 45,334.13 | 41,577.63 | 132.54 | 22.39 | 77.62 | 10.28 | 89.72 |
| Standard error (*SEM*) | 1,017.71 | 8,594.05 | 8,824.71 | 17.02 | 6.32 | 6.32 | 3.74 | 3.74 |
| Median | 2,850.5 | 34,424.5 | 29,551 | 113.55 | 15.80 | 84.20 | 6.93 | 93.07 |
| Mode | N/A | N/A | N/A | N/A | N/A | N/A | N/A | N/A |
| Standard deviation (*SD*) | 2,878.53 | 24,307.65 | 24,960.04 | 48.15 | 17.87 | 17.87 | 10.59 | 10.59 |
| Sample variance | 8,285,906 | 590,861,652.1 | 623,003,330 | 2,318.20 | 319.29 | 319.29 | 112.15 | 112.15 |
| Kurtosis | -0.44 | -1.51 | -1.55 | -1.57 | -0.10 | -0.10 | -0.30 | -0.30 |
| Skewness | 0.75 | 0.61 | 0.70 | 0.56 | 1.14 | -1.14 | 1.14 | -1.14 |
| Range | 8,257 | 64,497 | 62,087 | 123.6 | 49.85 | 49.85 | 27.73 | 27.73 |
| Minimum | 0 | 18,002 | 18,002 | 80.5 | 3.59 | 46.56 | 0 | 72.27 |
| Maximum | 8,257 | 82,499 | 80,089 | 204.1 | 53.44 | 96.41 | 27.73 | 100 |
| Sum | 30,052 | 362,673 | 332,621 | 1,060.3 | 179.08 | 620.92 | 82.25 | 717.75 |
| Count | 8 | 8 | 8 | 8 | 8 | 8 | 8 | 8 |
| Confidence level (*CI*) (95 %) | 2,406.5071 | 20,321.70 | 20,867.11 | 40.25 | 14.94 | 14.94 | 8.85 | 8.85 |
| Upper *CI* (95 %) | 6,163.01 | 65,655.83 | 62,444.74 | 172.79 | 37.32 | 92.55 | 19.13 | 98.57 |
| Lower *CI* (95 %) | 1,349.99 | 25,012.42 | 20,710.51 | 92.29 | 7.45 | 62.68 | 1.43 | 80.87 |
| *CS-HF = Handsewn sufficient small intestinal end-to-end anastomoses using simple continuous suture technique, tested in the high-flow model; mmHg = Millimeters of mercury; msec = Milliseconds; % = Percent; N/A = Not available.* | | | | | | | | |

**3. References**

[1] T. Berndt, ""Optmierung des Fluidkreislaufs für eine druckgeregelte Perfusionsstimulation im Bioreaktor". Semesterarbeit," Department of Orthopaedics and Sport Orthopaedics, Klinikum rechts der Isar, Technical University of Munich, Munich, 2020.

[2] A. Hangleiter, ""Weiterentwicklung eines Perfusionsbioreaktors und Realisierung eines druckprofilgeregelten Durchströmungsmodus (Tissue Engineering)". Masterarbeit," Department of Orthopaedics and Sport Orthopaedics, Klinikum rechts der Isar, Technical University of Munich, Munich, 2022.

[3] C. Micheler, ""Entwicklung eines automatisierten, druckgeregelten Perfusionsbioreaktors zur Kultivierung von 3D-Zellkulturen". Masterarbeit," Department of Orthopaedics and Sport Orthopaedics, Klinikum rechts der Isar, Technische Universität München Munich, 2017.

[4] C. Micheler, P. Foehr, and R. Burgkart, "Integration eines Low-Cost Touchscreen Display als Bedienoberfläche für das myRIO-System," 01/01 2017.

[5] C. Micheler *et al.*, "Bioreactor design for the mechanical stimulation by compression of 3D cell cultures," *Current Directions in Biomedical Engineering,* vol. 7, pp. 899-902, 10/09 2021, doi: 10.1515/cdbme-2021-2229.
